# The tumor microenvironment in leukemia: molecular pathways of immune evasion

**DOI:** 10.3389/fimmu.2026.1743920

**Published:** 2026-02-06

**Authors:** Ying Zhu, Jinying Liu, Fang Qiu, Zhongjian You, Zhirui Liu, Jiqiang Zeng, Jinqiong Zhong, Ziling Song, Shanrong Zhang, Jiawei Lu, Yujie Jiang, Jianshuo Liu, Zhimin Yan, Chen Lu

**Affiliations:** 1 Department of Blood Transfusion, First Affiliated Hospital of Gannan Medical University, Ganzhou, Jiangxi, China; 2 School of Medical Technology, Gannan Medical University, Ganzhou, Jiangxi, China; 3 Basic Medical College of Gannan Medical University, Ganzhou, Jiangxi, China; 4 Institute of Hematology & Blood Diseases Hospital, Chinese Academy of Medical Sciences & Peking Union Medical College, Beijing, China; 5 Department of Laboratory Medicine, First Affiliated Hospital of Gannan Medical University, Ganzhou, Jiangxi, China; 6 The First Clinical Medical School of Gannan Medical University, Ganzhou, Jiangxi, China; 7 Department of Hematology, First Affiliated Hospital of Gannan Medical University, Ganzhou, Jiangxi, China

**Keywords:** bone marrow niche, immune evasion, immunotherapy, leukemia, T-cell exhaustion, therapeutic resistance, tumor microenvironment

## Abstract

The interplay between the immune system and leukemia presents major challenges to effective therapy development. This Review examines mechanisms of immune evasion across leukemia subtypes, emphasizing T-cell exhaustion, regulatory T cells (Tregs), and antigen-presentation deficits. Globally, leukemia remains a significant burden, with approximately 460,000 new cases and 320,000 deaths estimated in 2021 alone. Recent studies reveal how the tumor microenvironment (TME) shapes immune behavior and how leukemic cells remodel it to support survival and therapeutic resistance. We illustrate these adaptive processes, highlighting the contributions of the bone-marrow niche and B-cell dysregulation in chronic lymphocytic leukemia (CLL). We further discuss implications for immunotherapy, noting that agents like magrolimab (anti-CD47) combined with azacitidine have demonstrated objective response rates (ORR) exceeding 80% in early-phase AML trials, though challenges such as on-target anemia persist. By integrating current evidence from preclinical metabolic profiling to Phase 3 clinical data on E-selectin inhibition (uproleselan)—we clarify the immune landscape of leukemia and outline avenues for innovative treatments. Ultimately, this Review underscores the need for multifaceted immunotherapeutic approaches that account for the complex interactions within the TME.

## 1 Introduction

Leukemias are heterogeneous hematological malignancies characterized by uncontrolled proliferation of abnormal hematopoietic cells in the bone marrow and peripheral blood. They are broadly classified into four major subtypes: acute myeloid leukemia (AML), acute lymphoblastic leukemia (ALL), chronic lymphocytic leukemia (CLL), and chronic myeloid leukemia (CML). Globally, leukemia remains a major health concern, with about 0.64 million new cases, 0.33 million deaths, and 11.66 million disability-adjusted life years (DALYs) in 2019 (1). By 2021, estimates reached 460–000 cases and 320–000 deaths (2). Although age-standardized incidence and mortality have declined over three decades, absolute case numbers are projected to rise by 2050 due to population growth (1, 2).

Epidemiologic patterns and survival differ markedly by subtype, age, sex, and region. AML, CML, and CLL predominantly affect older adults, whereas ALL is largely pediatric (1, 2). Males generally experience a higher leukemia burden compared to females (1, 2). In Australasia and Oceania, AML and non-Hodgkin lymphoma accounted for the largest leukemia/lymphoma burden between 2010 and 2019 (3). Mortality trends diverge—CML and ALL deaths have declined in India, while AML and CLL mortality increased (4). Outcomes for older adults remain poor: one- and five-year survival for AML patients aged 75–84 years were 18.2% and 2.7%, respectively (5). Despite improved pediatric survival, older individuals and some racial groups, including African American patients, have derived limited benefit (6).

Therapeutic advances remain constrained by toxicity, resistance, and relapse. In AML, intensive chemotherapy, venetoclax combinations, and mutation-targeted inhibitors (e.g., IDH, FLT3), plus allogeneic hematopoietic stem-cell transplantation (allo-HSCT), form the backbone of therapy but are limited by age-related toxicity and resistance. Access to novel drugs also varies regionally (3). In ALL, frontline chemotherapy with tyrosine kinase inhibitors (TKIs) for Philadelphia chromosome–positive (Ph+) disease achieves high remission rates, yet adults with minimal residual disease (MRD) positivity frequently relapse (7). Emerging immunotherapies—blinatumomab, inotuzumab ozogamicin, and CD19-targeted CAR T-cells—show promise but carry risks such as antigen escape, cytokine-release syndrome (CRS), and immune-effector-cell neurotoxicity (ICANS) (7, 8). While CAR T-cell therapy can induce durable remissions, particularly in pediatric patients, adult outcomes lag, and issues like suboptimal CAR-T persistence and the need for bridging therapies or subsequent consolidation approaches underscore the complexity of managing relapsed/refractory B-ALL (7, 9). Prior exposure to certain therapies, such as inotuzumab ozogamicin, can also influence post-CAR T-cell survival outcomes (10). For CLL, Bruton’s tyrosine kinase (BTK) inhibitors (both covalent and noncovalent) and BCL2 inhibitors, often in time-limited combinations, have revolutionized treatment. Nevertheless, resistance mechanisms, including BTK C481S mutations, intolerance, and the risk of infections or Richter transformation, remain significant challenges (11). Finally, in CML, tyrosine kinase inhibitors (TKIs), including newer agents like asciminib, have transformed the disease into a manageable chronic condition for many. Yet, a substantial minority of patients experience treatment failure due to resistance or toxicity, and the risk of progression to blast crisis persists (12). While treatment-free remission (TFR) is an increasingly feasible objective for patients achieving a sustained deep molecular response, it is not universally achievable, and the optimal duration of TKI consolidation to maximize TFR success is still under investigation (13).

The intricate interplay between leukemia cells and their surrounding bone marrow tumor microenvironment (TME) is increasingly recognized as a critical determinant of disease progression, therapeutic response, and relapse (14, 15). While many studies emphasize epidemiology and clinical outcomes, fewer characterize the bone-marrow TME’s architecture, cellular composition (e.g., mesenchymal stromal, endothelial, osteolineage, adipocytic, macrophage, dendritic, NK, regulatory T, and effector T cells) (16, 17), key signaling axes (CXCL12–CXCR4, VLA-4–VCAM-1)) (18), or its contrasts with solid-tumor TMEs (19). Understanding dynamic interactions—adhesion, exosomes, tunneling nanotubes, gap junctions, metabolic coupling, and niche-mediated drug tolerance—is essential (20, 21).

Given the persistent challenges in achieving durable remissions and overcoming therapeutic resistance across leukemia subtypes, a deeper understanding of the bone marrow TME is imperative (22). This review adopts a bone marrow niche-centric lens to systematically organize and synthesize current knowledge regarding immune evasion mechanisms within the TME, encompassing both innate and adaptive immune arms (23). We highlight immunometabolic regulation of leukemia–TME interactions and the impact of spatial and multi-omics technologies—including single-cell sequencing, spatial transcriptomics/proteomics, ATAC-seq, CyTOF, and imaging mass cytometry—that now enable high-resolution dissection (24, 25). We also examine bidirectional interactions between the TME and therapeutic modalities—chemotherapy, TKIs, BCL2 inhibitors (venetoclax), bispecific antibodies, CAR T-cells, checkpoint blockade, and HSCT (26, 27). By integrating these strands, we aim to identify novel vulnerabilities in the leukemia–TME axis and propose rational co-targeting strategies to enhance efficacy and outcomes (28, 29).

### 1.1 Search strategy and selection criteria

To provide a comprehensive overview of immune evasion in the leukemic bone marrow niche, we conducted a narrative review of the literature using PubMed and Web of Science databases. The search focused on peer-reviewed articles published between January 2010 and November 2025, with a priority on studies from the last five years to capture recent advances in single-cell omics and immunotherapy. Key search terms included “Leukemia,” “Tumor Microenvironment,” “Bone Marrow Niche,” “Immune Evasion,” “T-cell Exhaustion,” “AML,” “ALL,” “CLL,” “CML,” and “Immunometabolism.” We included pivotal preclinical studies, observational patient cohorts, and interventional clinical trials, excluding conference abstracts lacking full peer-reviewed data.

## 2 Architecture of the leukemic bone−marrow niche

The bone marrow (BM) microenvironment, or “niche,” is a complex ecosystem essential for hematopoiesis. In leukemia, this niche is hijacked and reprogrammed to sustain malignant cells, promote immune evasion, and confer therapeutic resistance. Understanding its cellular architecture and signaling crosstalk is crucial for developing effective therapies.

### 2.1 Cellular and extracellular matrix components

The leukemic BM niche consists of diverse stromal, vascular, and immune cells embedded in an extracellular matrix (ECM) that supports leukemic growth. Mesenchymal stromal cells (MSCs) are central multipotent regulators that differentiate into adipocytes and osteoblasts. In the tumor microenvironment (TME), MSCs can adopt cancer-associated fibroblast (CAF)–like states induced by leukemic signals; for example, miR-21 mediates human bone-marrow MSC transition via exosomes (30). These CAF-like states can be induced by leukemic cells, as seen in head and neck squamous cell carcinoma (HNSCC) where microRNA-21 (miR-21) mediates the transition of human bone marrow mesenchymal stem cells (hBMSCs) to CAFs via exosomes (31). Within the marrow, MSCs localize to perivascular and endosteal regions, and CXCL12-abundant reticular (CAR) fibroblasts display transcriptional polarizations including inflammatory, peri-arteriolar, peri-sinusoidal, and adipocytic phenotypes (32).

Leukemia drives diverse MSC phenotypes that reinforce malignancy. Aggressive B-ALL subtypes enrich MSC cytokine secretion—IL-6, IL-8, CCL2, and MIF—enhancing leukemic fitness and chemoresistance (33). Leukemic cells also alter MSC bioelectric properties, depolarizing membranes and downregulating CaV1.2 L-type Ca^2+^ channels; CaV1.2 restoration partly reverses these effects, partly through tunneling-nanotube transfer (34, 35). AML blasts further skew MSCs toward osteoblastic differentiation, conferring drug resistance via Wnt-pathway dysregulation critical for leukemia-stem-cell (LSC) maintenance (36, 37). They also hijack mitochondria from stromal cells through CD38-dependent transfer to fuel oxidative phosphorylation (38).

Endothelial and osteolineage cells contribute to vascular remodeling. Leukemia induces endothelial clonal expansion via apelin signaling, promoting disease in acute erythroid leukemia (39–41). These endothelial cells secrete angiocrine factors such as CXCL12 and VCAM-1 that sustain B-ALL survival (42). Osteoblasts are similarly reprogrammed by leukemic cells to reinforce drug resistance (36, 43).

Adipocytes, particularly in extramedullary adipose tissue (EMAT), contribute to the ALL microenvironment and are gaining recognition as drug testing systems due to emerging evidence linking obesity to poor prognosis (44). While specific lipid shuttling and fatty-acid oxidation (FAO) support mechanisms (e.g., via CD36, CPT1A) are not explicitly detailed in the provided contexts, the presence of adipocytic CAR cells suggests their potential involvement in metabolic support within the niche (32).

The innate immune compartment becomes immunosuppressive. In CML, increases in CD11b^+^Ly6C^int PMN-MDSCs, CD11b^+^Ly6C^high M-MDSCs, and F4/80^+^ macrophages promote immune suppression via PD-L1 and Arg1 upregulation (45). AML environments feature leukemia-associated macrophages (LAMs) alongside Tregs and Bregs (46). In CLL, monocytes differentiate into M2-like nurse-like cells (NLCs) expressing IRF4, IDO, CD163, and CD206, which secrete IL-6 and nurture leukemic cells (47). Synthetic STING agonists can re-polarize MDSCs and M2 macrophages by suppressing c-Myc signaling (48).

Adaptive immune subsets, including natural killer (NK) cells, T lymphocytes, and B lymphocytes, also exhibit dysfunction. In AML, patients harbor clonally expanded terminal effector memory CD45RA+ (TEMRA) CD8 T cells and abundant immunosuppressive CD4 Tregs in the marrow. These Tregs suppress CD8 effector activity, and AML blasts express T cell inhibitory molecules such as TIGIT ligands, CD244, and VISTA (49). In CLL, low oxygen levels in the niche decrease T-cell proliferation, promote glycolysis, and lead to PD-1+ and IL-10-secreting T cells (47). Myelodysplastic syndromes (MDS) also show active remodeling of the adaptive immune system, with the emergence of immunologically active niches containing T, B, and plasmacytoid dendritic cells (pDCs), and clonally expanded memory CD8 T cells (43).

The extracellular matrix (ECM) undergoes marked remodeling that regulates cell adhesion, signaling, and mechanotransduction (50). Fibronectin, collagens, and laminins engage receptors such as VLA-4 (α4β1) and VCAM-1, mediating stromal adhesion and NF-κB–dependent chemoresistance (51, 52). Matrix stiffening enhances malignant progression (50). ndothelial E-selectin facilitates leukemic dormancy and survival (53). Gap junctions (GJs) formed by connexins allow exchange of ions, metabolites, and even organelles that bolster leukemia proliferation (54). Exosomes and tunneling nanotubes also mediate intercellular communication, as in miR-21–driven CAF induction and CaV1.2 transfer (31, 34).

### 2.2 Adhesion and retention axes

Leukemic retention within BM niches relies on adhesion and trafficking pathways co-opted from normal hematopoiesis. The CXCL12–CXCR4 axis anchors leukemic cells to perivascular stroma, maintaining dormancy and survival (55). In CLL, NEDD9 modulates CXCR4-CXCL12–driven migration; Nedd9 deficiency delays disease by reducing CXCR4 expression and tissue infiltration (56). CXCL12–CXCR4/CXCR7 signaling also promotes proliferation, angiogenesis, and metastasis across cancers (55). CXCR4 antagonists such as plerixafor mobilize blasts from the protective niche to enhance chemotherapy sensitivity. FLT3 inhibitors may upregulate CXCR4 and E-selectin ligands via ERK suppression, supporting combination strategies (57).

VLA-4–VCAM-1 and integrin–selectin interactions are also vital for leukemic cell retention. VLA-4 binding to VCAM-1 or fibronectin regulates hematopoietic progenitor migration and survival, and in leukemia activates NF-κB signaling to drive chemoresistance (51, 52).

E-selectin, a vascular adhesion molecule expressed on endothelium, recognizes sialyl Lewis X (sLeX) ligands on cancer cells. E-selectin, a vascular adhesion molecule expressed on endothelium, recognizes sialyl Lewis X (sLeX) ligands enriched on leukemic cells, particularly in B-ALL, facilitating adhesion, dormancy, and therapy resistance (53, 58). The E-selectin antagonist uproleselan (GMI-1271) disrupts these interactions, mobilizing blasts and enhancing chemotherapy efficacy (58).

Leukemia dissemination also depends on trafficking cues and extramedullary sanctuaries. Cdc42 inhibition impairs LSC self-renewal and mobilizes blasts from the marrow (59). Nedd9 loss limits CLL homing to secondary organs (56). Extramedullary sites such as adipose tissue in ALL serve as therapy-evasive niches (44). Involvement of the spleen, liver, CNS, skin, and testes has major clinical implications for relapse.

### 2.3 Leukemia−specific remodeling

Leukemic cells actively remodel their microenvironment, transforming a supportive hematopoietic niche into a sanctuary that promotes their survival, proliferation, and resistance to therapy. This stromal reprogramming by leukemic blasts involves complex cytokine/chemokine rewiring and metabolic crosstalk. As noted, aggressive B-ALL subtypes induce MSCs to secrete pro-leukemic cytokines like IL-6, IL-8, CCL2, and MIF, which enhance leukemic cell fitness and chemoresistance (33). In AML, the transforming growth factor beta 1 (TGFβ1)/SMAD2/SMAD4 signaling pathway is critical for upregulating IL3Rα (CD123) on LSCs, skewing progenitors toward inflammatory myelopoiesis and representing a potential target for LSC-directed therapies (60). Metabolic crosstalk is also evident, with AML blasts hijacking mitochondria from BMSCs to fuel oxidative phosphorylation (38). In CLL, the hypoxic niche leads to increased adenosine generation and signaling through the A2A receptor, affecting tumor and host cellular responses, including IL-10 production in lymphocytes and IL-6 in myeloid cells, and promoting M2-like macrophage differentiation that enhances nurturing properties (47). Leukemic cells also prime stromal cells towards an osteoblast lineage, dysregulating Wnt signaling and promoting drug resistance (36).

Leukemia also induces antigen-presentation alterations, PD-L1 upregulation, and suppressive myeloid programs. In CML, BCR-ABL1 activity directly regulates immunosuppressive genes like Arg1 in myeloid-derived suppressor cells (MDSCs) and leads to PD-L1 upregulation in M-MDSCs/macrophages, contributing to immune evasion (45). In AML, leukemic blasts drive impaired T cell immunity, leading to distinct T cell compositions with expanded TEMRA CD8 T cells and abundant immunosuppressive Tregs. AML blasts also exhibit increased expression of T cell inhibitory molecules such as TIGIT ligands, CD244, and VISTA, which restrain anti-tumor T cell immunity (49). Immunotherapy with pembrolizumab and decitabine in refractory/relapsed AML can lead to global or local enrichment of immune cells proximal to leukemia cells and potential alterations in ligand-receptor signaling, such as TWEAK, which may correlate with clinical responses (61).

Angiocrine cytokine and growth-factor signaling from endothelial cells is co-opted by leukemia. Endothelial cells form interconnected vasculature networks in AML organoids and show significant remodeling in the leukemic niche. In FLT3-ITD+ acute myeloid leukemia (AML), the bone marrow niche undergoes substantial vascular changes during disease progression. Specifically, there is a loss of arterioles and a corresponding increase in sinusoids. These alterations are driven by tumor necrosis factor alpha (TNFα), a cytokine produced by AML blasts. TNFα induces the downregulation of microRNA-126 (miR-126) in endothelial cells (ECs), leading to a depletion of CD31+Sca-1high ECs (associated with arterioles) and an increase in CD31+Sca-1low ECs (associated with sinusoids) (62). This loss of miR-126-rich ECs reduces the supply of miR-126 to leukemic stem cells (LSCs), which in turn promotes LSC entry into the cell cycle and leukemia growth (62). Conversely, anti-leukemic treatments, such as tyrosine kinase inhibitors (TKIs), can decrease TNFα production by blasts, leading to an increase in miR-126 high ECs and an enhanced supply of miR-126 to LSCs. However, high miR-126 levels can paradoxically safeguard LSCs, contributing to more severe disease in secondary transplantation models. Therefore, therapeutic deprivation of EC miR-126 could overcome this treatment-induced LSC protection by preventing the re-vascularization of CD31+Sca-1high ECs (62). Beyond these specific molecular changes, the overall disruption of the bone marrow microenvironment by AML cells, including its structural and cellular components, plays a crucial role in disease progression and protects AML blasts from therapeutic agents (63). In preleukemic stages of AML, there are already observed decreases in normal endothelial cell populations, such as Cdh5+ ECs, indicating early remodeling of the niche (64, 65). Endothelial cells, along with other stromal and immune cells, actively interact with leukemic blasts through deregulated molecular pathways, influencing leukemia development, survival, chemoresistance, and migratory properties (66).

## 3 Innate immune evasion

Leukemia cells employ diverse mechanisms to evade innate immune recognition and elimination, which is critical for disease persistence, resistance, and relapse. These strategies are often linked to the bone marrow (BM) microenvironment, where leukemic cells interact with stromal and immune cells to foster an immunosuppressive niche that shields them from host defenses and therapeutic interventions (67, 68). Understanding these complex interactions is paramount for developing novel treatment strategies.

### 3.1 NK cell escape

Natural killer (NK) cells are crucial innate immune components capable of lysing malignant cells without prior sensitization. Leukemic cells circumvent this surveillance through sophisticated mechanisms, primarily by shedding NKG2D ligands (e.g., MICA/B, ULBPs). These ligands typically trigger NK cell cytotoxicity (69), however, tumor cells release soluble forms via metalloprotease-mediated cleavage (ADAM10, ADAM17). This shedding leads to the down-modulation of NKG2D on NK cells, impairing their ability to recognize and kill leukemic blasts (70). Strategies to restore NK cell function include preventing shedding (e.g., ADAM17 inhibition) or using adoptive NK cell therapies/engagers. Leukemic cells also exploit non-classical human leukocyte antigen (HLA) upregulation, specifically HLA-E and HLA-G, to inhibit NK cell activity. HLA-E interacts with the inhibitory receptor CD94/NKG2A on NK cells, delivering a “stop” signal that prevents lysis. HLA-G binds to inhibitory receptors LILRB1 (ILT2) and LILRB2 (ILT4) on NK cells and myeloid cells, respectively, contributing to an immunosuppressive milieu. Therapeutic interventions target this axis with NKG2A blockade (e.g., monalizumab) to release the inhibitory brake on NK cells.

A critical evasion mechanism involves the CD47–signal regulatory protein alpha (SIRPα) “don’t eat me” signaling axis. CD47 is overexpressed on many cancer cells, including leukemic blasts, shielding them from macrophage phagocytosis by binding to SIRPα on phagocytic cells (71, 72). In AML and high-risk MDS, disruption of this axis has progressed to clinical evaluation (71). Agents like magrolimab (anti-CD47) combined with azacitidine have demonstrated efficacy in Phase 1b trials, although on-target anemia remains a manageable adverse event (72). Conversely, in CLL, while CD47 expression correlates with disease progression, clinical efficacy of monotherapy has been more modest, suggesting a need for combination strategies (73).

### 3.2 Myeloid rewiring

In Acute Myeloid Leukemia (AML), the bone marrow microenvironment undergoes significant myeloid rewiring, leading to the expansion of myeloid-derived suppressor cells (MDSCs) and tumor-associated macrophages (TAMs). These cells act as key orchestrators of immunosuppression. MDSCs accumulate in the AML niche and promote tumor growth (74), through diverse suppressive mechanisms. These mechanisms include the depletion of essential amino acids via arginase-1/2 (ARG1/2), the production of reactive nitrogen and oxygen species (iNOS/NO, ROS), and the secretion of immunosuppressive cytokines such as IL-10 and TGF-β. Furthermore, catabolism of tryptophan via IDO1, adenosine production (CD39/CD73), and metabolic reprogramming toward an M2-like TAM phenotype contribute to this suppressive milieu. Preclinical studies highlight the potential of targeting these pathways; for instance, mutations in PPARα correlate with chemoresistance, while SHP-2 ablation in tumor-bearing mice prevents MDSC accumulation, thereby enhancing effector cell differentiation and T-cell function (74, 75). Additionally, the JAK-STAT pathway, particularly involving leptin (LEP) and STAT1, is upregulated in AML-derived MSCs, supporting leukemic cell survival (75).

In Chronic Lymphocytic Leukemia (CLL) and other B-cell malignancies, complement regulatory proteins represent a distinct evasion mechanism. Proteins such as CD55 and CD59 are often overexpressed to shield leukemic blasts from complement-dependent cytotoxicity (CDC), a key effector mechanism of monoclonal antibody therapies like rituximab. By inhibiting the membrane attack complex, CD55 and CD59 reduce antibody-mediated complement activation (76). Strategies currently being explored to overcome this resistance include the down-modulation of these regulatory proteins or the combination of therapeutic antibodies with agents designed to enhance complement activation (77).

### 3.3 Pattern-recognition and danger signaling

Leukemic cells can manipulate pattern-recognition receptor (PRR) signaling, especially via Toll-like receptors (TLRs), to their advantage. TLRs are expressed on leukemic blasts, stromal, and myeloid cells, recognizing PAMPs (from infections) and DAMPs (e.g., HMGB1, S100A8/A9) (78). TLR modulation can be bidirectional. In AML, TLR2, TLR4, and TLR9 are often expressed. Stimulation with TLR2/4 ligands or IFN-γ can induce B7-H1 (PD-L1) expression on AML blasts, protecting them from cytotoxic T-cell lysis (78). Upregulation of the TLR pathway in AML-derived MSCs (via increased TLR2) suggests an enhanced inflammatory environment that contributes to progression (75).

The STING (stimulator of interferon genes) pathway detects cytosolic DNA, activating anti-tumor immunity via type I interferons. Leukemic cells can suppress this pathway through epigenetic silencing of cGAS/STING or upregulation of enzymes (e.g., ENPP1, TREX1) that degrade DNA. Such suppression compromises type I interferon signaling, impairs antigen presentation, and dampens NK/myeloid activation, facilitating evasion. Therapeutic intersections involve small-molecule STING agonists to restore type I interferon responses and anti-tumor immunity.

## 4 Adaptive immune evasion

Adaptive immune evasion remains a major obstacle to durable leukemia control, as malignant cells evolve mechanisms to avoid immune recognition and elimination. These processes intertwine with the bone-marrow niche, which fosters an immunosuppressive milieu that limits therapeutic efficacy. Understanding these pathways is essential for refining checkpoint blockade, bispecific antibodies, CAR T-cell therapies, and hematopoietic stem-cell transplantation (HSCT) (79, 80).

### 4.1 T-cell exhaustion

T-cell exhaustion is a dysfunctional state caused by chronic antigen exposure, marked by reduced effector function, impaired proliferation, and sustained expression of inhibitory receptors (81, 82). These inhibitory receptors, often referred to as immune checkpoints (ICs), play a crucial role in dampening T-cell responses. Key inhibitory axes include programmed cell death protein 1 (PD-1) and its ligands PD-L1 and PD-L2, cytotoxic T-lymphocyte-associated protein 4 (CTLA-4), T-cell immunoglobulin and mucin domain-containing protein 3 (TIM-3), lymphocyte-activation gene 3 (LAG-3), and T-cell immunoreceptor with Ig and ITIM domains (TIGIT) (81, 83–88). Upon ligand binding, these receptors suppresses T-cell receptor (TCR) signaling, reprograms metabolism from oxidative phosphorylation to glycolysis, and diminishes cytokine and cytotoxic activity (81). For instance, protein tyrosine phosphatases PTPN2 and PTPN1 can reduce JAK and STAT1/3/5 signaling, thereby abrogating T-cell receptor and cytokine responses, a pathway that can be targeted to augment anti-tumor immunity (87).

The expression patterns of these checkpoint receptors vary across leukemias and T-cell subsets. In acute myeloid leukemia (AML), T-cells often exhibit an exhausted phenotype, marked by high expression of PD-1, TIGIT, LAG-3, and TIM-3 (87). TIM-3 is also frequently found on AML blast cells; its negativity is linked to better overall survival in certain AML subgroups (89). Similarly, increased PD-L1 expression in AML correlates with worse overall survival, especially in patients with Flt3-ITD mutations (89).

In chronic myeloid leukemia (CML), the immune system, including natural killer (NK) cells, can show signs of exhaustion with upregulated HAVCR2 (encoding TIM-3) and TIGIT expression, particularly after tyrosine kinase inhibitor (TKI) cessation and subsequent relapse (90). While specific data on T-cell exhaustion in acute lymphoblastic leukemia (ALL) from the provided research papers are limited, the general principle of chronic antigen exposure leading to exhaustion applies across malignancies. Chronic lymphocytic leukemia (CLL) T-cells exhibit a multi-checkpoint exhausted phenotype. This mirrors observations in other chronic immune challenges, like HIV-tuberculosis co-infection, where increased co-expression of multiple immune checkpoint molecules (TIM-3, CTLA-4, LAG-3) on both CD4+ and CD8+ T-cells is seen, correlating with disease progression (86). This co-expression suggests a gradient of exhaustion severity, engaging multiple inhibitory pathways simultaneously.

Therapeutic strategies targeting these immune checkpoints show promise, albeit with specific limitations in leukemias. Hypomethylating agents (HMAs) like decitabine enhance the PD-1 pathway in myelodysplastic syndromes (MDS) and AML, providing a strong rationale for combination with PD-1/PD-L1 inhibitors (88). The anti-PD-L1 antibody avelumab combined with decitabine in unfit AML patients was safe and tolerable. It led to the upregulation of activation markers (e.g., CD69, CD226, CD28, ICOS, 4-1BB) and the downregulation of inhibitory molecules (e.g., TIGIT, TIM-3, CD160, LAG-3, 2B4, BTLA) on CD8+ T-cells, enhancing anti-leukemia immunity (88). HMAs can also upregulate checkpoint receptors and ligands, potentially sensitizing leukemic cells to blockade. For example, atezolizumab (anti-PD-L1) combined with magrolimab (anti-CD47) is hypothesized to enhance T-cell anti-tumor responses in relapsed/refractory AML by reversing T-cell exhaustion (91). Beyond PD-1/PD-L1, TIM-3 blockade (e.g., sabatolimab) is being investigated in MDS/AML, often with HMAs. Similarly, strategies for LAG-3 and TIGIT blockade, frequently combined with PD-1/PD-L1 inhibitors, aim to overcome the multi-checkpoint expression characteristic of deeply exhausted T-cells, especially in CLL. Targeted therapies like TKIs in CML also modulate immune function, with imatinib and dasatinib showing differential off-target effects on immune effector cells (92).

**Figure 1:**
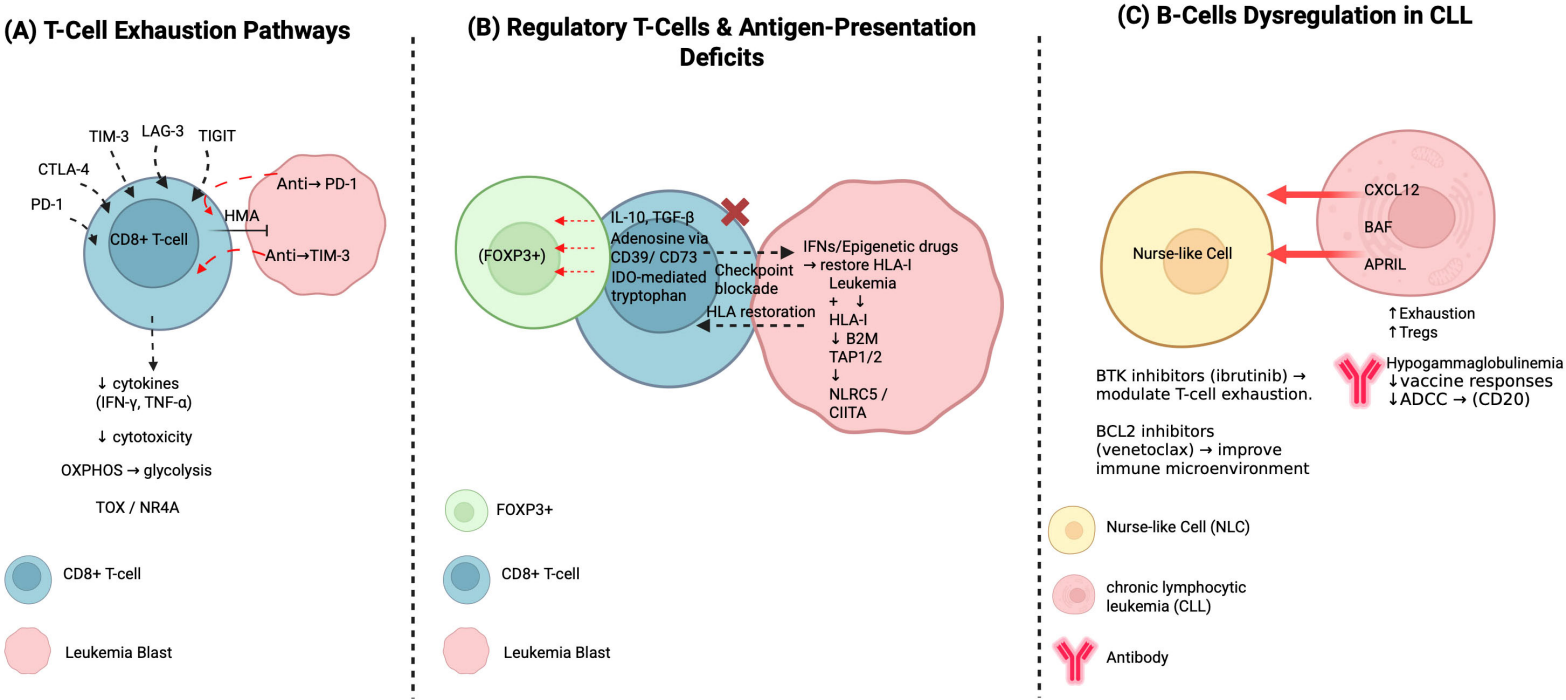
Mechanisms of T-cell exhaustion in leukemia: a schematic overview. This figure summarizes adaptive immune evasion mechanisms in leukemia. **(A)** T-cell exhaustion is driven by chronic antigen stimulation and checkpoint signaling, resulting in metabolic and transcriptional reprogramming that dampens cytotoxic activity. **(B)** Regulatory T cells and leukemic cells collaborate with the bone marrow microenvironment to suppress immunity via inhibitory cytokines, adenosine metabolism, tryptophan depletion, and defects in antigen presentation machinery, culminating in immune escape. **(C)** In CLL, nurse-like cells and altered B-cell interactions reinforce T-cell exhaustion and regulatory pathways, while defective humoral immunity undermines antibody-based responses. PD-1, Programmed cell death protein 1; CTLA-4, Cytotoxic T-lymphocyte-associated protein 4; TIM-3, T-cell immunoglobulin and mucin-domain containing-3; LAG-3, Lymphocyte activation gene 3; TIGIT, T cell immunoreceptor with Ig and ITIM domains; CD8+ T cell, Cluster of Differentiation 8 positive T cell; HMA, Hypomethylating agent; IFN gamma, Interferon gamma; TNF alpha, Tumor necrosis factor alpha; OXPHOS, Oxidative phosphorylation; TOX, Thymocyte selection-associated high mobility group box protein; NR4A, Nuclear receptor subfamily 4 group A; IL10, Interleukin 10; TGF beta, Transforming growth factor beta; CD39, Cluster of Differentiation 39; CD73, Cluster of Differentiation 73; IDO, Indoleamine 2,3-dioxygenase mediated tryptophan metabolism; FOXP3, Forkhead box P3; CLL, Chronic lymphocytic leukemia; NKC, Natural killer cell; BAF, BRG1-associated factor; CXCL12, C-X-C motif chemokine ligand 12; APRIL, A proliferation-inducing ligand; Treg, Regulatory T cell; ADCC, Antibody-dependent cellular cytotoxicity; CD20, Cluster of Differentiation 20; BTK inhibitor, Bruton’s tyrosine kinase inhibitor; BCL2 inhibitor, B-cell lymphoma 2 inhibitor.

The sustained expression and dysfunctional state of exhausted T-cells are governed by a distinct transcriptional program. Key regulators like TOX and the NR4A family are critical in establishing and maintaining this exhausted epigenetic landscape (93, 94). OX drives T-cell differentiation towards exhaustion, promoting chromatin accessibility at exhaustion-specific gene loci (93, 94). NR4A family members, induced by chronic TCR stimulation, repress effector genes (95, 96). This makes a crucial distinction between reversible T-cell “dysfunction” and a more entrenched, epigenetically fixed exhausted state (96, 97). Disease-specific transcriptional landscapes are evident: chronic antigen drive in CLL leads to profound multi-checkpoint exhaustion, while AML checkpoint expression is altered by treatment-induced phenotypes (97, 98). These profiles link to clinical outcomes, with deeper exhaustion correlating with poorer prognosis (97, 98). Emerging strategies to reprogram exhaustion include epigenetic priming, cytokine support, and metabolic modulation, aiming to reverse epigenetic modifications and restore T-cell function (96, 97). **Figure 1** illustrates the pathways of T-cell exhaustion that contribute to immune dysfunction and therapeutic resistance in leukemia.

### 4.2 Regulatory T cells and antigen-presentation deficits

Regulatory T cells (Tregs), characterized by FOXP3+ within the CD4+ T-cell subset, are potent immunosuppressive cells vital for maintaining immune tolerance. In leukemia, Tregs are often expanded and enriched in the bone marrow and peripheral blood, significantly contributing to the immunosuppressive tumor microenvironment (99). Their suppressive mechanisms are multifaceted: they secrete inhibitory cytokines (IL-10, TGF-β) that inhibit effector T-cell proliferation and function (99). Tregs also use the adenosine pathway, where surface ectonucleotidases CD39 and CD73 convert ATP/ADP to adenosine, a potent immunosuppressive molecule (99, 100). Furthermore, indoleamine 2,3-dioxygenase (IDO)-mediated tryptophan depletion by myeloid cells, often induced by Tregs, suppresses cytotoxic T-cell responses by creating a local essential amino acid deficiency (99). This crosstalk within the bone marrow niche fosters a tolerogenic milieu that impairs cytotoxic T-cell priming and persistence (99).

Leukemia therapies significantly alter Treg frequency and function. TKIs in CML modulate immune cell populations, including Tregs (100). Similarly, BTK and BCL2 inhibitors (e.g., venetoclax) impact Treg dynamics, with some studies suggesting a reduction in numbers or altered suppressive capacity, potentially contributing to anti-leukemic effects. HMAs also influence Treg populations, with complex, context-dependent effects (99). Downregulation or loss of HLA class I expression is a common immune escape mechanism in various leukemias (AML, ALL, CLL, CML) (100). This can result from mutations or loss of β2-microglobulin (B2M), a crucial component, or defects in the antigen processing machinery (TAP1/2, NLRC5, CIITA) (99). B2M mutations or loss are particularly relevant under immune pressure, such as after T-cell redirecting therapies (99). This phenomenon is a hallmark of immunoediting, where highly immunogenic clones are eliminated, selecting for less immunogenic variants with antigen presentation defects (99). This greatly impacts CTL surveillance and is a recognized mechanism of relapse after potent immunotherapies (CAR-T cell therapy, bispecific antibodies), where antigen or HLA loss renders cells invisible to engineered T-cells (99). Therapeutically, strategies to upregulate HLA expression (e.g., with interferons or epigenetic modifiers) are being evaluated to restore presentation and enhance T-cell-based immunotherapies (99). Combining these with checkpoint blockade could overcome both T-cell exhaustion and antigen presentation deficits. However, in the context of hematopoietic stem cell transplantation (HSCT), enhancing HLA expression risks exacerbating graft-versus-host disease (GVHD) while augmenting graft-versus-leukemia (GVL) effects, requiring careful balance (99).

### 4.3 B-cell dysregulation in CLL

CLL presents a unique landscape of adaptive immune evasion, heavily influenced by the dysregulated B-cell compartment and its microenvironment. Nurse-like cells (NLCs) are critical components of the CLL bone marrow and lymph node microenvironment (101, 102). These stromal cells provide essential supportive cues, including chemokines (CXCL12) and survival factors (BAFF, APRIL) (103–107). These factors promote CLL cell survival and proliferation within specialized “proliferation centers,” crucial for disease progression (106–109). NLCs and other stromal elements also foster a profoundly tolerogenic milieu that reinforces T-cell exhaustion and promotes regulatory T cell expansion, further dampening anti-leukemic immunity (109). NLC supportive signals can also impair T-cell help and disrupt normal follicular architecture, contributing to overall immune dysfunction (110).

A hallmark of CLL is profound impaired humoral immunity, manifesting as hypogammaglobulinemia, defective class-switch recombination, and vaccine hyporesponsiveness, leading to increased susceptibility to infections (110–113). This humoral dysfunction is intrinsic to the disease and contributes to immune evasion (112, 113). Targeted therapies like BTK inhibitors (e.g., ibrutinib) and BCL2 inhibitors (e.g., venetoclax) have revolutionized CLL treatment (114–117). While primarily targeting leukemic B-cells, these agents can partially restore or further impair humoral and T-cell function. BTK inhibitors can improve T-cell function by reducing exhaustion markers and enhancing proliferation but may also exacerbate hypogammaglobulinemia (118–123). BCL2 inhibitors induce CLL cell apoptosis and can indirectly improve the immune microenvironment (124). The impaired humoral immunity in CLL also impacts the efficacy of antibody-based therapies, such as anti-CD20 monoclonal antibodies, by affecting complement activation and antibody-dependent cellular cytotoxicity, though they remain a cornerstone of treatment (77, 125, 126).

## 5 Immunometabolism and hypoxia

**Figure 2:**
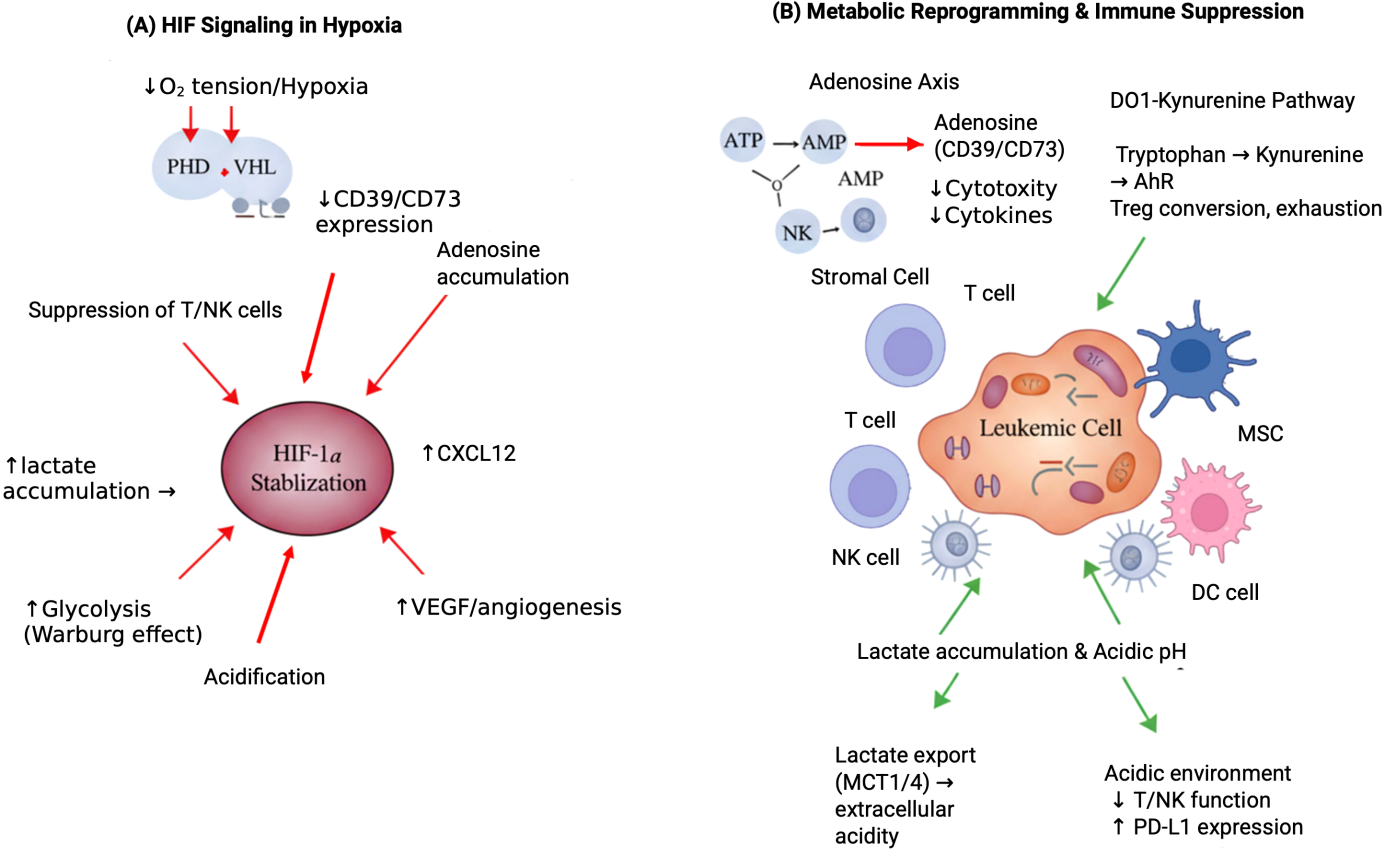
Hypoxia-driven immunometabolic reprogramming in the leukemic niche. This figure illustrates the impact of hypoxia on metabolic checkpoints and immunometabolic reprogramming within the leukemic bone marrow niche. **(A)** Hypoxia stabilizes HIF-1α through PHD/VHL inhibition, promoting glycolysis, lactate accumulation, acidification, VEGF/angiogenesis, CXCL12 expression, and CD39/CD73-driven adenosine buildup, collectively suppressing T/NK cell function. **(B)** Metabolic checkpoints in the leukemic niche—adenosine signaling, the IDO1–kynurenine pathway, and lactate export—further impair T/NK cell activity by reducing cytotoxicity, driving Treg conversion, inducing exhaustion, and enhancing PD-L1 expression, thereby supporting leukemic cell survival. HIF-1α, Hypoxia-Inducible Factor 1-alpha, PHD, Prolyl Hydroxylase Domain, VHL, von Hippel-Lindau, VEGF, Vascular Endothelial Growth Factor, CXCL12, C-X-C Motif Chemokine Ligand 12, CD39, Cluster of Differentiation 39, CD73, Cluster of Differentiation 73, NK, Natural Killer cells, IDO1, Indoleamine 2,3-dioxygenase 1, PD-L1, Programmed Death-Ligand 1, Treg, Regulatory T cells; MSC cells, Mesenchymal Stem Cells.

The bone marrow microenvironment’s metabolic stress and fluctuating oxygen tension profoundly influence immune cell function, contribute to leukemia persistence, and dictate therapeutic responses. Within this niche, aberrant metabolite gradients and hypoxic conditions can bias effector immune cells (T and NK cells) towards an exhausted or suppressive phenotype, while supporting leukemic cell survival and proliferation. Understanding these immunometabolic adaptations is crucial for effective anti-leukemia strategies. **Figure 2** provides a schematic overview of how these metabolic checkpoints, often exacerbated by hypoxia, facilitate immune evasion and support leukemic cell survival.

### 5.1 Metabolic checkpoints

#### 5.1.1 Adenosine axis

The adenosine axis represents a critical immunosuppressive pathway in the tumor microenvironment (TME), including leukemias. Extracellular adenosine triphosphate (ATP) is sequentially hydrolyzed to adenosine monophosphate (AMP) and then to adenosine by ectonucleotidases CD39 (also known as ENTPD1) and CD73 (also known as NT5E), respectively (127, 128). These enzymes are expressed on leukemic blasts, stromal, and myeloid cells (128–130). Adenosine engages G-protein–coupled A2A (A2AR) and A2B (A2BR) receptors on immune cells, elevating intracellular cAMP and protein kinase A (PKA) activity. This suppresses T-cell receptor signaling, reduces NK cell cytotoxicity, and limits cytokine production, facilitating immune evasion (128, 131).

Hypoxia and inflammatory cues, often mediated by hypoxia-inducible factor 1-alpha (HIF-1α), upregulate CD39 and CD73, amplifying adenosine-mediated suppression (128). In multiple myeloma (MM), elevated bone marrow adenosine and blockade of CD39, CD73, or A2AR (e.g., AZD4635) restore immune activation, increase IFN-γ production, and reduce tumor burden in mice, paralleling PD-1/PD-L1 inhibition (128). In juvenile myelomonocytic leukemia (JMML), high CD39/CD73 expression suppresses T-cell function, reversible by CD39 inhibition (130). In acute myeloid leukemia (AML), chemotherapy agents such as daunorubicin and cytarabine induce CD39/CD73 on dendritic cells, stabilizing regulatory T-cell (Treg) phenotypes (132, 133). Therapeutic strategies include A2AR antagonists, anti-CD73 antibodies, and triple antagonists like CT3021 (A1/A2A/A2B), which show activity in adenosine-rich TMEs (131). Dual A2A/A2B antagonists (e.g., M1069) also demonstrate enhanced anti-tumor efficacy by preventing A2BR compensation (134).

#### 5.1.2 IDO1–Kynurenine pathway

The indoleamine-2,3-dioxygenase-1 (IDO1)–kynurenine pathway represents another critical metabolic checkpoint promoting immune tolerance. IDO1 catalyzes tryptophan degradation into kynurenine, which activates the aryl hydrocarbon receptor (AhR) on T cells, promoting exhaustion, Treg polarization, and NK cell dysfunction (135, 136). IDO1 is expressed by leukemic blasts, dendritic, and myeloid cells, and is inducible by inflammatory mediators such as IFN-γ (137, 138).

In AML, IFN-γ–producing leukemic cells induce IDO1 in mesenchymal stromal cells (MSCs), driving Treg expansion in an IDO1-dependent manner (137, 138). This fosters immune tolerance and disease progression. Preclinical work highlights dynamic modulation of the IDO–kynurenine–AhR axis after therapy, informing rational combinations with immune checkpoint blockade (ICB) (135). Although IDO1 inhibitors faced setbacks in solid tumors, their potential in hematologic malignancies—possibly via epigenetic priming or combinatorial strategies—remains under investigation to overcome prior efficacy and safety limitations.

#### 5.1.3 Arginine depletion via ARG1/ARG2

Arginine depletion, driven by arginase activity, constitutes another immunosuppressive mechanism. Myeloid-derived suppressor cells (MDSCs) and tumor-associated macrophages (TAMs) express arginase-1 (ARG1) and arginase-2 (ARG2), depleting extracellular L-arginine essential for T-cell proliferation and NK cytotoxicity (139). This leads to CD3ζ downregulation and impaired immune function. Cationic amino acid transporters (CATs; e.g., CAT-1, CAT-2A, CAT-2B) regulate arginine uptake and availability in the TME (140).

In chronic myeloid leukemia (CML), diagnostic MDSCs display elevated ARG1 activity, generating an immunotolerant milieu that suppresses anti-tumor T cells. Both MDSC expansion and ARG1 activity decline following imatinib therapy, underscoring their role in immune escape (141). Increased arginase activity correlates with advanced disease and poor prognosis. Therapeutic approaches include arginase inhibitors, arginine supplementation, or precursors to restore arginine levels, often combined with PD-1/PD-L1 blockade to enhance immune responses. Development of next-generation arginase inhibitors with improved pharmacokinetics is ongoing for hematologic applications (139).

### 5.1.4 Lactate accumulation, acidic pH, and HIF-1α stabilization

Leukemic cells exhibit enhanced aerobic glycolysis (the Warburg effect), leading to excessive lactate accumulation in the TME (142). Lactate export occurs via monocarboxylate transporters MCT1 (SLC16A1) and MCT4 (SLC16A3), while carbonic anhydrases (e.g., CAIX) regulate pH (142, 143). The resulting acidic milieu (pH < 6.9) impairs T and NK cell function, antigen presentation, and promotes PD-L1 expression, fostering immune escape (142, 144).

Hypoxia, characteristic of the bone marrow niche, stabilizes HIF-1α by inhibiting prolyl hydroxylase (PHD)–mediated degradation via von Hippel-Lindau (VHL) protein. Stabilized HIF-1α reprograms metabolism, enhances glycolysis, and modulates chemokine and adhesion networks such as CXCL12, supporting leukemic survival (142, 145). In AML, leukemic stem cells (LSCs) uniquely depend on oxidative phosphorylation (OXPHOS) and fatty acid oxidation (FAO) even within hypoxic niches, contrasting with bulk blasts that show high glycolysis and MCT4 expression. This metabolic flexibility renders LSCs vulnerable to metabolic disruption. Conversely, in Acute Lymphoblastic Leukemia (ALL), hypoxia-induced HIF-1α mediates glucocorticoid resistance, underscoring context-dependent metabolic adaptations across leukemia subtypes (146–148).

Therapeutically, strategies to target these metabolic dependencies are advancing. MCT1 inhibition with agents like AZD3965 has demonstrated the ability to reduce lactate export and sensitize cells to chemotherapy in preclinical models and early-phase clinical studies (149, 150). Buffering strategies to neutralize TME acidity enhance immunotherapy efficacy (151, 152). Similarly, targeting LDHA or CAIX disrupts this axis and has been shown to restore immune activity *in vitro*. Buffering strategies to neutralize TME acidity have also been validated in preclinical settings to enhance immunotherapy efficacy (149, 153, 154).

### 5.2 Organelle and metabolite exchange

#### 5.2.1 Tunneling nanotubes and extracellular vesicles

Intercellular communication in the leukemic bone marrow niche extends beyond soluble factors to involve direct physical connections and vesicle-mediated transfer. Tunneling nanotubes (TNTs) are actin-rich conduits that enable the direct intercellular transfer of components, including organelles (155–157). Extracellular vesicles (EVs), such as exosomes and microvesicles, act as crucial carriers of enzymes, microRNAs (miRNAs), and immunomodulators, facilitating communication among leukemic, stromal, and immune cells (158–160).

In AML, Mesenchymal stromal cells (MSCs) donate mitochondria and metabolites to leukemic blasts via TNTs and EVs. This transfer enhances leukemic cells’ oxidative phosphorylation (OXPHOS) capacity, improves redox buffering, and contributes to drug tolerance, specifically venetoclax resistance (161–165). Regulators like Miro1 and connexins are key to this exchange (166). Furthermore, leukemic EVs actively remodel the immune and stromal compartments (167–170). or instance, leukemic EVs can upregulate CD39/CD73 on recipient cells, skew myeloid cells toward an immunosuppressive phenotype, and blunt NK/T cell cytotoxic function, thus promoting immune evasion (171–174).

Therapeutic concepts aim to disrupt these pathways, though they remain largely in preclinical development. Tools like the exosome inhibitor GW4869 or modulation of the mitochondrial adaptor protein Miro1 have been investigated in murine models to inhibit TNT-mediated transfer and EV biogenesis/uptake (166, 175). While conventional agents (HMAs, venetoclax) may intersect with mitochondrial metabolism, their precise impact on these transfer mechanisms is still being defined (162–165).

#### 5.2.2 Redox control and mitochondrial transfer

Leukemic cells employ sophisticated redox adaptations to survive in the challenging bone marrow microenvironment. These involve activating Nrf2-driven antioxidant programs and maintaining glutathione/NADPH balance, along with intricate mitochondrial reactive oxygen species (ROS) signaling (176–179). These redox imbalances significantly impair immune synapse formation and the cytotoxic function of T and NK cells, contributing to immune evasion (172–174, 179).

A critical aspect of redox control in AML is the transfer of healthy mitochondria from MSCs and endothelial cells to AML cells (161). This acquisition enhances AML cells’ OXPHOS capacity, making them more resilient to metabolic stress and contributing to venetoclax resistance and overall survival (161–165). Conversely, increased intracellular ROS can sometimes sensitize leukemic blasts to therapy by inducing apoptosis or enhancing immune recognition (165, 179). This complex interplay means the balance of ROS and antioxidant defenses dictates therapeutic vulnerability.

Targeted strategies are being developed to exploit these vulnerabilities. These include complex I inhibition, blockade of fatty acid oxidation (FAO), or direct redox modulation to restore immune cell function and sensitize leukemic cells (180–183). Targeting mitochondrial complex I, for instance, has shown promise in preclinical AML models by disrupting OXPHOS and inducing apoptosis in LSCs (181–183). While these strategies are in early development, careful consideration of safety signals and off-target effects on healthy hematopoietic cells is paramount.

## 6 Signaling crosstalk driving evasion and resistance

**Figure 3:**
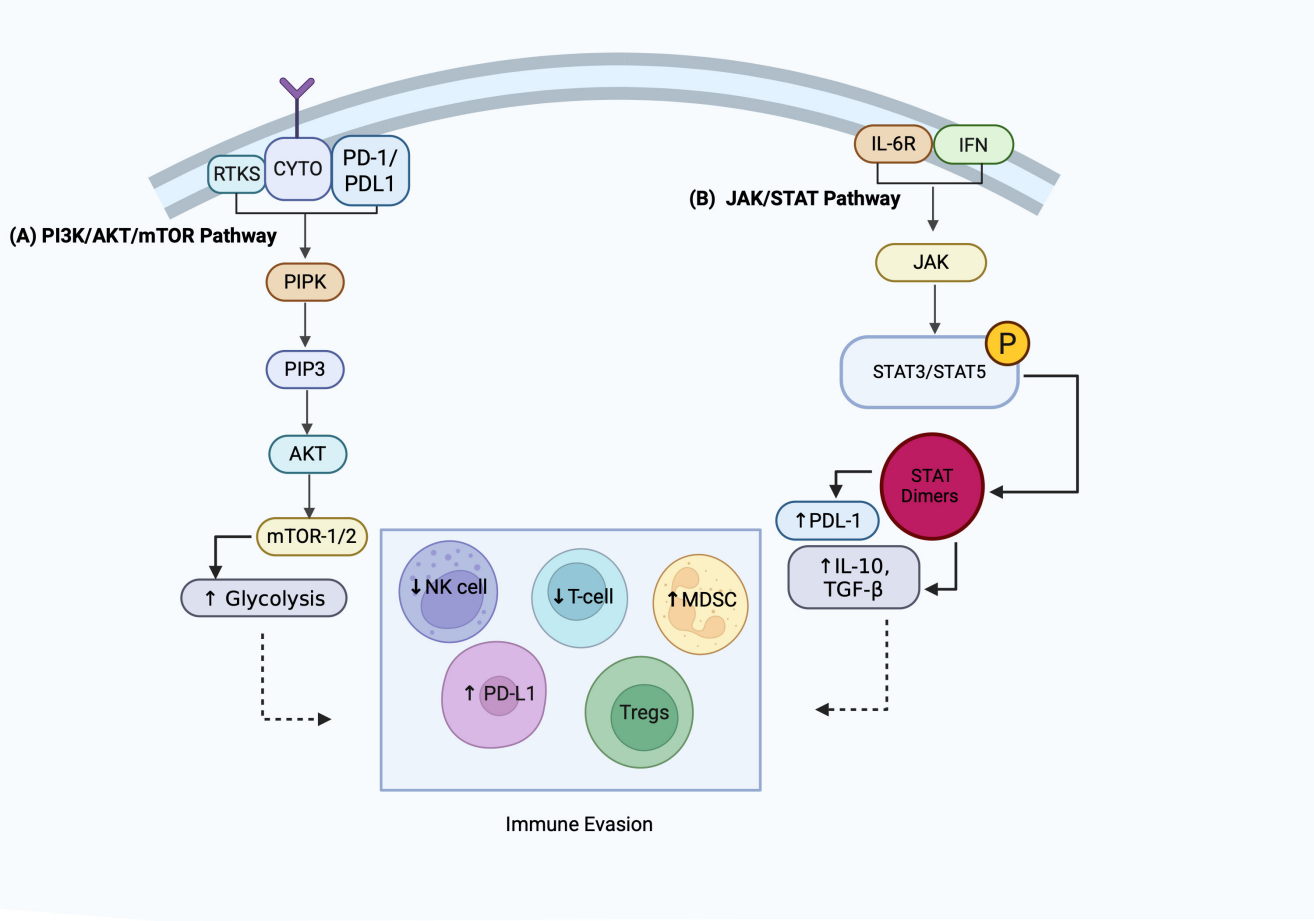
Signaling crosstalk driving immune evasion in the leukemic niche. This figure highlights two key signaling pathways that mediate immune suppression in leukemia. **(A)** The PI3K/AKT/mTOR pathway enhances glycolysis and promotes PD-L1 upregulation, contributing to reduced NK and T cell activity and expansion of regulatory T cells (Tregs) and myeloid-derived suppressor cells (MDSCs). **(B)** The JAK/STAT pathway, activated by cytokines such as IL-6 and interferons, induces STAT3/STAT5 dimerization, leading to increased PD-L1 expression and secretion of immunosuppressive cytokines (IL-10, TGF-β), thereby reinforcing immune evasion. PI3K, Phosphoinositide 3-kinase, AKT, Protein kinase B, mTOR, Mechanistic target of rapamycin, PD-L1, Programmed death-ligand 1, NK, Natural killer, Tregs, Regulatory T cells, MDSCs, Myeloid-derived suppressor cells, JAK, Janus kinase, STAT, Signal transducer and activator of transcription, IL-6, Interleukin-6, IL-10, Interleukin-10, TGF-β, Transforming growth factor-beta; PIPK, Phosphatidylinositol Phosphate Kinase; PIP3, Phosphatidylinositol (3,4,5)-trisphosphate; NK cells, Natural Killer cells; T-cells, T lymphocytes; PDL-1, Programmed Death-Ligand 1; MDSC, Myeloid-Derived Suppressor Cells; STAT3, Signal Transducer and Activator of Transcription 3; STAT5, Signal Transducer and Activator of Transcription 5; IL-6R, Interleukin 6 Receptor; IFN, Interferon.

Signaling crosstalk between leukemic cells and the surrounding bone marrow microenvironment (TME) is a central driver of immune evasion, drug tolerance, and disease persistence. This intricate network integrates niche architecture, innate/adaptive immune evasion, and immunometabolic adaptations to orchestrate a pro-tumorigenic environment that shields malignant cells from therapeutic intervention and immune surveillance. **Figure 3** schematically represents how key pathways integrate microenvironmental cues to establish this network, driving both immune evasion and therapeutic resistance.

### 6.1 Key pathways

Several key signaling pathways are frequently dysregulated and interconnected within the leukemic TME, promoting cancer cell survival, proliferation, trafficking, and immune escape. The PI3K/AKT/mTOR, JAK/STAT, and NF-κB pathways are pivotal in this crosstalk (184–191). tromal, endothelial, and immune-derived cytokines (e.g., IL-6, TNF-α), along with BCR or TCR engagement, converge on these pathways. For instance, Galectin-3 (Gal-3) upregulation in the bone marrow microenvironment (BMME) promotes AML cell adhesion and survival via PI3K/AKT/mTOR, Ras/Raf/MEK/ERK, JAK/STAT, and NF-κB, leading to chemotherapy resistance and relapse. Similarly, tumor-derived extracellular vesicles (TDEVs) modulate NF-κB to promote inflammation/immune evasion and orchestrate PI3K/AKT/mTOR signaling to abrogate immune responses and drive proliferation (186). The immune checkpoint molecule B7-H3 (CD276) also signals through JAK/STAT, NF-κB, PI3K/Akt, and MAPK pathways, driving tumor growth, invasion, and apoptosis inhibition while promoting immune evasion (187).

The outputs of these pathways directly link to immune evasion phenotypes. STAT3 and NF-κB activation can induce PD-L1 and IDO1 expression, contributing to immune suppression. Metabolic rewiring, often driven by mTOR and HIF, supports the adenosine axis (CD39/CD73) and contributes to an immunosuppressive milieu (185, 189). STAT3 activation also plays a role in the polarization of myeloid-derived suppressor cells (MDSCs) and tumor-associated macrophages (TAMs) (192). Adhesion-mediated drug resistance is frequently linked to PI3K/AKT signaling downstream of microenvironmental interactions (193).

Disease-specific anchors underscore the importance of these pathways. In AML, FLT3-ITD mutations often cause constitutive STAT5 activation (193). Chronic BCR signaling in chronic lymphocytic leukemia (CLL) drives PI3Kδ and NF-κB activation, crucial for cell survival and proliferation within protective niches (194). BCR-ABL1 signaling in chronic myeloid leukemia (CML) primes PI3K and STAT networks. In glioblastoma stem cells (GSCs), Notch, Wnt/β-catenin, and Hedgehog pathways are critical regulators of maintenance, plasticity, and immune evasion (195).

Beyond these, the Notch, Wnt, and Hedgehog (HH) pathways also mediate TME crosstalk. Notch ligand engagement(e.g., JAG1/DLL) by stromal cells promotes context-specific survival/immune modulation, with NOTCH1 mutations significant in T-ALL (191, 196). Wnt/β-catenin signaling is crucial for cancer stem cell (CSC) stemness, quiescence, and immune evasion, including repression of antigen presentation. Osteolineage and stromal Wnt cues are important in AML (197–200). Hedgehog signaling, mediated via SMO/GLI from stromal ligands (SHH/IHH/DHH), is linked to leukemic stem cell (LSC) maintenance and therapy tolerance (195, 196, 200). Hedgehog signaling (via SMO/GLI) is linked to leukemic stem cell (LSC) maintenance and therapy tolerance.

Chemokine axes, including CXCL/CXCR and S1P/S1PR, regulate leukemic cell trafficking and niche interactions. The TME’s role in promoting adhesion and survival involves these general pathways (193). CXCR5-CXCL13 is implicated in CLL node homing, and CCR7-CCL19/21 in CNS/nodal trafficking in ALL, facilitating sanctuary formation and resistance (194). S1P-S1PR1 gradients control egress, circulation, and access to protective niches, influencing therapy response.

### 6.2 Intrinsic vs. microenvironment−induced programs

The interplay between leukemia-intrinsic mutations and TME cues creates a robust system for immune evasion and drug resistance. Oncogenic drivers, such as FLT3-ITD and NPM1 mutations in AML, BCR-ABL1 in CML, and chronic BCR/BTK/PI3Kδ signaling or NOTCH1 mutations in CLL and T-ALL, sensitize specific signaling nodes to TME ligands (193, 195, 198). This sensitization amplifies downstream STAT3, NF-κB, and PI3K outputs, enforcing immune-evasion programs like PD-L1, CD47, IDO1, and CD39/CD73 expression (185–187). Hypoxia, a common feature of the TME, further amplifies these pathways through HIF-1α, stabilizing evasion and survival states, particularly in CSCs (185, 192, 200). Hypoxia sustains the self-renewal characteristics of cancer cells and modulates pathways that confer epithelial-to-mesenchymal transition (EMT) characteristics, contributing to tumor progression and resistance (192).

Furthermore, bidirectional stromal feedback loops maintain the leukemic niche. Leukemic cells release cytokines/EVs that activate stromal NF-κB, STAT3, Notch, Wnt, and Hedgehog programs (200–202). In turn, activated stromal cells provide essential support (e.g., chemokines like CXCL12, cytokines IL-6/BAFF, and metabolic resources). This support enhances leukemic cell adhesion, quiescence, and immune suppression (192, 193, 200, 203). For example, reciprocal signaling in AML induces Gal-3 expression, promoting AML cell adhesion/survival and chemotherapy resistance. Targeted agents (e.g., BTK and PI3Kδ inhibitors, JAK inhibitors, and CXCR4 antagonists) can disrupt these loops, but their effects are often context-dependent, necessitating combination therapies to overcome resistance (188, 191, 197, 198).

## 7 Spatial, single−cell, and multi−omics views

The advent of spatial and single-cell technologies has significantly transformed the understanding of leukemic bone marrow (BM) ecosystems. These advanced methods reveal the intricate organization of niches, diverse states of immune cells, and critical interactions influencing therapeutic responses—insights previously obscured by bulk assays. By dissecting cellular heterogeneity and spatial context, these technologies offer a nuanced view of the leukemic microenvironment, showing how leukemic stem cells (LSCs) interact with their surroundings and how these interactions can be therapeutically targeted.

### 7.1 Spatial heterogeneity

Current BM models delineate two primary niches: the endosteal niche (proximal to osteolineage cells) and the perivascular niche (associated with sinusoidal/arteriolar structures). The endosteal niche often supports quiescent hematopoietic stem cells (HSCs), while the perivascular niche favors proliferative states (204) In leukemia, these niches remodel significantly. Acute myeloid leukemia (AML) cells disrupt spatial organization via mechanisms like CXCL12 remodeling, altering LSC retention/migration and vascular permeability (62, 63). LSC localization and dormancy remain controversial; two-photon microscopy suggests LSCs exploit peri-arteriolar hypoxic conditions for quiescence in AML, while other reports suggest perisinusoidal residence (204, 205). In chronic myeloid leukemia (CML), LSCs persist in protective vascular and endosteal niches, contributing to tyrosine kinase inhibitor (TKI) resistance (206, 207).

Beyond niche zoning, the BM microenvironment is defined by hypoxia- and metabolite-driven microdomains. Spatial gradients of oxygen, pH, and metabolites (e.g., lactate, adenosine) create immunosuppressive microenvironments influencing leukemic progression (63). Hypoxia-inducible factor 1-alpha (HIF-1α) programs are upregulated in these microdomains, promoting the expression of monocarboxylate transporters (MCT1/MCT4) and carbonic anhydrases (e.g., CAIX), which aid leukemic cell adaptation (63). These microdomains often co-localize with specific stromal/immune cells (e.g., M2-like macrophages, MDSCs), further exacerbating immune evasion and predicting therapy response or sanctuary behavior (208–210). For example, AML LSCs residing in hypoxia-linked quiescent niches may show venetoclax response correlating with their reliance on OXPHOS (211). In chronic lymphocytic leukemia (CLL), proliferation centers in lymph nodes are characteristic, receiving pro-survival/proliferative stimuli from the microenvironment (212–214).

### 7.2 Single−cell and multi−omics mapping

Single-cell RNA sequencing (scRNA-seq) and T-cell receptor sequencing (TCR-seq) provide profound insights into leukemic/stromal compartment cellular composition and functional states, revealing the heterogeneity of MSCs, endothelial subtypes, and osteolineage cells (204, 215). T-cell and NK-cell exhaustion trajectories have been mapped in leukemias, demonstrating co-expression of inhibitory receptors (PD-1, TIM-3, LAG-3, TIGIT) associated with dysfunctional immune responses (216, 217). Transcriptional programs driven by TOX and NR4A transcription factors are implicated in these states. Tools like RNA velocity and pseudotime analysis infer lineage dynamics and transitions from cytotoxic to exhausted states, offering crucial insights into how therapies (HMAs, TKIs) reshape the immune landscape across AML, ALL, CLL, and CML (217).

**Figure 4:**
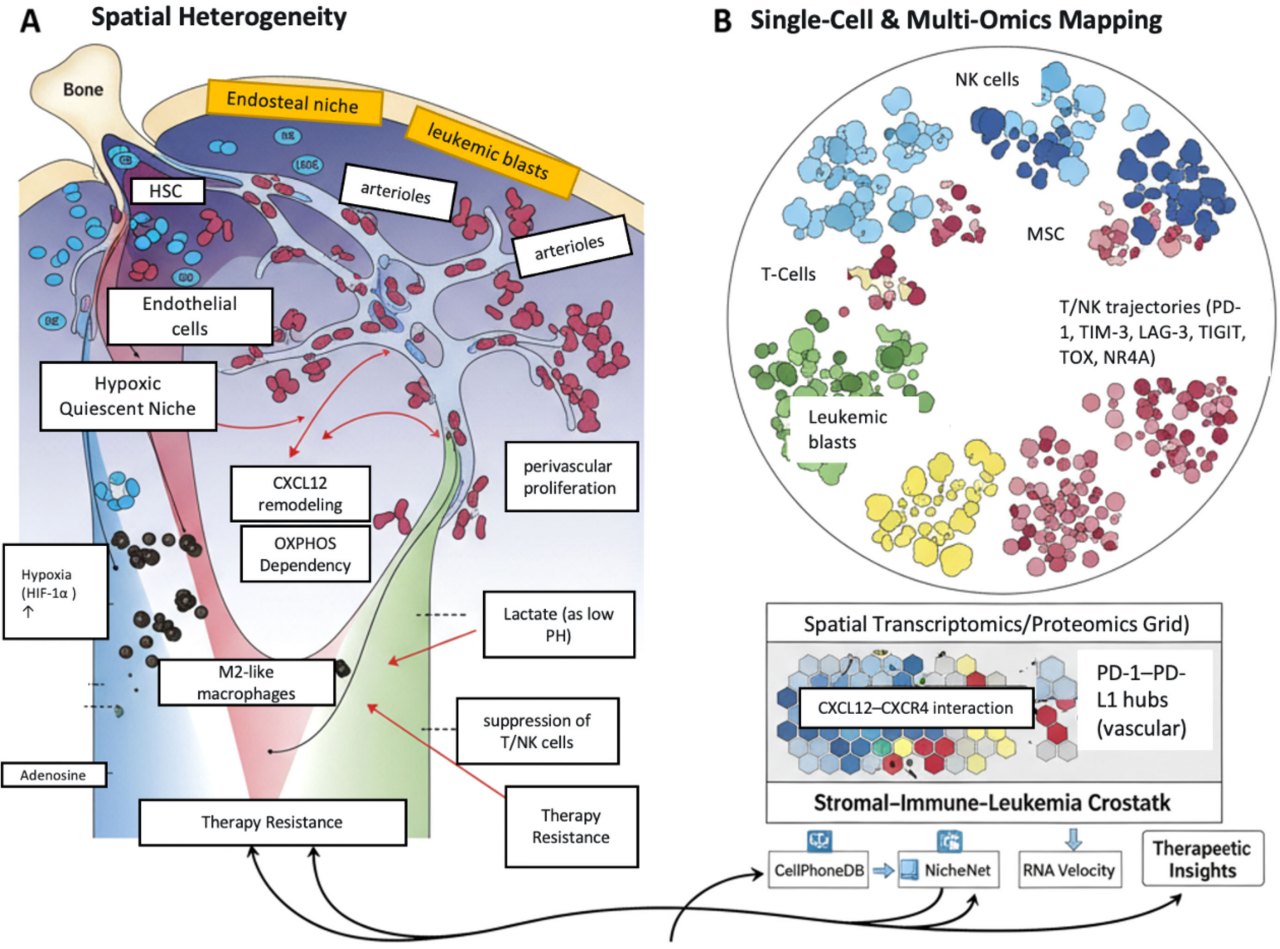
Multi-omics deconstruction of the leukemic bone marrow niche. This figure illustrates how integrating spatial and single-cell technologies provides a multi-scale view of the leukemic microenvironment. **(A)** Architectural Remodeling and Immunosuppressive Microdomains: This panel depict the remodeling of the primary bone marrow niches (endosteal and perivascular) by leukemia. It highlights the formation of hypoxia- and metabolite-driven gradients that create immunosuppressive microdomains, fostering leukemic stem cell (LSC) persistence and immune suppression. **(B)** Mapping Cellular States and Interactions: This panel conceptualizes the outputs of single-cell and multi-omics profiling. These technologies reveal T-cell exhaustion trajectories, stromal cell heterogeneity, and the precise spatial locations of key ligand-receptor hubs (e.g., CXCL12–CXCR4, PD-1–PD-L1, and adenosine) that govern cell behavior and therapeutic response. LSC, Leukemic Stem Cell, PD-1, Programmed Death-1, PD-L1, Programmed Death-Ligand 1, CXCL12, C-X-C Motif Chemokine Ligand 12, CXCR4, C-X-C Motif Chemokine Receptor 4; NK cells, Natural Killer cells; MSC, Mesenchymal Stem Cells; T cells, T lymphocytes; PD1, Programmed cell death protein 1; PDL-1, Programmed death-ligand 1.

Spatial transcriptomics (ST) and proteomics further enhance understanding by mapping ligand-receptor interactions and stromal cues. Modalities (e.g., 10x Visium, MERFISH, CosMx SMI) and spatial proteomics (e.g., IMC, CODEX) identify interaction neighborhoods, such as CXCL12-CXCR4 and PD-1-PD-L1 (204, 208, 218). Biomarker patterns—including LSC proximity to arterioles, PD-L1-rich vascular niches, and CD39/CD73-adenosine hubs—have been associated with minimal residual disease, relapse, or resistance to venetoclax/chemotherapy (206, 211). Computational frameworks (CellPhoneDB, NicheNet) facilitate data integration with spatial context (218). However, technical caveats (resolution limits, decalcification artifacts, batch effects) must be acknowledged as they affect interpretation (43). **Figure 4** synthesizes these concepts, illustrating how spatial and multi-omics technologies map the complex cellular/molecular architecture of the leukemic BM, depicting key niches, interactions, and immunosuppressive microdomains influencing therapeutic outcomes.

## 8 Clinical translation and therapeutics

The bone marrow tumor microenvironment (TME) profoundly influences therapeutic response, resistance, and relapse in hematologic malignancies. Understanding the interplay between leukemic cells and their niche—encompassing adhesion, trafficking, immune suppression, and metabolic reprogramming—is crucial for effective clinical strategies. This section explores how conventional agents, immunotherapies, and niche-directed drugs intersect with these TME-driven mechanisms to shape patient outcomes.

### 8.1 Conventional therapy–TME interactions

Conventional therapies for hematologic malignancies are increasingly understood through their interactions with the TME, revealing mechanisms of both efficacy and resistance. Hypomethylating agents (HMAs), such as azacitidine and decitabine, exert immunomodulatory effects by up-regulating HLA class I and II molecules and antigen-processing genes, which can enhance tumor cell recognition by the immune system. While they may variably induce programmed death-ligand 1 (PD-L1) expression, HMAs can also influence dendritic cells and myeloid-derived suppressor cells (MDSCs), potentially re-priming exhausted T cells to restore anti-tumor immunity (219). Beyond direct cellular effects, HMAs can reshape the stromal compartment by altering CXCL12 expression and adhesion molecule profiles, thereby influencing immune cell function and drug response.

Venetoclax (VEN)-based regimens have transformed the treatment landscape for acute myeloid leukemia (AML), particularly by targeting the strong dependency of AML cells on mitochondrial oxidative phosphorylation (OXPHOS) for their increased proliferation (220). However, a subset of AML cells can survive through metabolic activation of fatty acid oxidation (FAO), which uncouples mitochondrial OXPHOS and contributes to chemoresistance. This metabolic rewiring, often facilitated by interactions with bone marrow stromal cells, enables drug-resistant AML cells and leukemic stem cells to acquire resistance against OXPHOS and FAO inhibitors (220). Microenvironmental protection also involves adhesion molecules like very late antigen-4 (VLA-4), C-X-C chemokine receptor type 4 (CXCR4), and E-selectin, which mediate leukemic cell retention within protective niches. Resistance mechanisms to VEN often involve these TME interactions, providing a rationale for combining VEN with FLT3 inhibitors (FLT3i), isocitrate dehydrogenase inhibitors (IDHi), or agents targeting CXCR4 or E-selectin, as well as metabolic modulators. Clinical considerations for VEN include hematologic toxicities and scheduling to optimize efficacy while managing adverse events.

FLT3 and IDH inhibitors represent targeted therapies with significant TME implications. FLT3i, used in FLT3-mutated AML, can affect cytokine circuits, including a surge in FLT3 ligand post-chemotherapy, and disrupt stromal protection mediated by the CXCL12–CXCR4 axis and PI3K signaling pathways (221). Emerging strategies combine FLT3i with VEN, HMAs, or CXCR4 antagonists to overcome resistance and enhance therapeutic depth. For instance, CXCR4 antagonists can mobilize leukemic cells from protective niches, potentially increasing their susceptibility to FLT3i and VEN (222). IDH1/2 inhibitors, such as ivosidenib and enasidenib, lower the oncometabolite 2-hydroxyglutarate (2-HG), promoting cellular differentiation in IDH-mutated AML. In intrahepatic cholangiocarcinoma (iCCA), IDH1/2 mutations are associated with a non-inflamed TME and downregulation of antigen processing and presentation machinery, suggesting that IDH1/2 inhibitors may restore DNA methylation and expression of molecules involved in antigen presentation, thereby improving the efficacy of immune checkpoint inhibitors (ICIs) (223). Combination strategies with VEN or HMAs are also being analyzed for IDHi to address TME-mediated resistance and enhance anti-leukemic activity.

### 8.2 Immunotherapies

Immunotherapies face significant TME challenges. CAR T-cell therapy encounters barriers (e.g., PD-L1, TGF-β, adenosine signaling, poor trafficking) that attenuate function, especially where T-cell fitness is compromised (e.g., CLL) (224–226). These barriers include immunosuppressive signaling pathways such as PD-L1, transforming growth factor-beta (TGF-β), and adenosine, as well as poor trafficking and retention imbalances of CAR T-cells within the marrow. Antigen loss or trogocytosis by tumor cells and the presence of myeloid-suppressive niches, often populated by MDSCs, further contribute to resistance (219, 224). Differences in co-stimulation domains, such as CD28 versus 4-1BB, influence CAR T-cell persistence and exhaustion profiles. Strategies to overcome these barriers include combining CAR T-cells with checkpoint inhibitors targeting PD-1, T-cell immunoglobulin and mucin domain-containing protein 3 (TIM-3), or NKG2A, or blocking adenosine, indoleamine 2,3-dioxygenase (IDO), or arginase (ARG) pathways (224). “Armored” CAR T-cells, engineered to secrete cytokines or chemokines, and retargeting CXCR4 or CXCR5 can improve homing and function. However, a critical caution for myeloid-antigen-targeted CAR T-cells is the potential for significant myelotoxicity due to on-target, off-tumor effects on healthy hematopoietic stem cells.

Bispecific T-cell engagers (BiTEs), like blinatumomab (CD3-CD19), show success in B acute lymphoblastic leukemia (B-ALL) but myeloid-targeted bispecifics face TME-driven T-cell dysfunction (227). Their short half-life can be overcome by innovative delivery (e.g., secretory BiTEs encoded in oncolytic viruses) (228). Concurrent checkpoint or metabolic modulation may enhance the efficacy of these agents by mitigating TME-induced suppression.

Immune checkpoint blockade (ICB) (PD-1/PD-L1, CTLA-4) shows limited monotherapy activity but excels in combination (e.g., HMAs in AML/MDS). Emerging checkpoints like TIM-3 (e.g., sabatolimab), LAG-3, and TIGIT are under investigation, often showing enhanced efficacy in combination strategies that achieve greater T-cell reinvigoration (229–232). Safety issues, including immune-related adverse events and GVHD risk post-HSCT, are critical (233).

### 8.3 Microenvironment-targeting agents

Targeting the TME directly offers a promising avenue to overcome resistance and enhance the efficacy of conventional and immune-based therapies. CXCR4 antagonists, such as plerixafor, disrupt the CXCL12–CXCR4 axis, which is critical for retaining leukemic blasts and leukemic stem cells (LSCs) within protective bone marrow niches. By mobilizing these cells into the peripheral circulation, CXCR4 antagonists can sensitize them to chemotherapy, venetoclax, or hypomethylating agents. Plerixafor is clinically established for hematopoietic stem cell mobilization, and its use in combination with granulocyte colony-stimulating factor (G-CSF) has shown high yields of CD34+ cells with primitive signatures for gene therapy applications (234, 235). Other oral CXCR4 antagonists, like mavorixafor, are in clinical development for conditions such as WHIM syndrome, demonstrating the ability to durably increase neutrophil and lymphocyte counts and reduce infection rates by addressing the underlying CXCR4 gain-of-function mutations (236, 237). While effective, hematologic and trafficking-related adverse events, including potential splenic enlargement with G-CSF, must be carefully managed (238).

E-selectin inhibitors represent another class of TME-targeting agents designed to block adhesion-mediated drug resistance and disrupt inflammatory vascular niches. Uproleselan (GMI-1271), a specific E-selectin antagonist, has shown promise in preclinical and clinical studies. It works by inhibiting cancer cell tethering, rolling, and extravasation, reducing adhesion, and mobilizing leukemic cells from protective niches, thereby increasing their susceptibility to chemotherapy. Clinical trials have demonstrated that uproleselan has a favorable safety profile and can improve the efficacy of chemotherapy while reducing side effects such as neutropenia and intestinal mucositis, which are common in AML treatment (58). A pivotal Phase 3 registration trial (NCT03616470) is evaluating uproleselan with standard salvage chemotherapy in relapsed/refractory (R/R) AML, with initial data indicating that high E-selectin ligand expression contributes to chemotherapy resistance and relapse, which can be reversed by E-selectin inhibition. This trial also reported a dramatic reduction in severe oral mucositis with uproleselan in combination with MEC chemotherapy, alongside promising overall survival and remission rates in earlier phases (239).

In AML and MDS, therapeutic blockade of the CD47 or SIRPα-Fc axis has advanced from the preclinical validation described in Section 3.1 to active clinical evaluation. Magrolimab (5F9), a first-in-class anti-CD47 antibody, has demonstrated robust activity in combination with azacitidine. Phase 1b data indicate high objective response rates and the potential to eradicate leukemic stem cells, particularly in high-risk TP53-mutant populations. To manage on-target anemia resulting from physiological CD47 expression on red blood cells, treatment protocols now incorporate a priming and intrapatient dose-escalation regimen. This strategy has successfully mitigated hematotoxicity, yielding a safety profile comparable to azacitidine monotherapy (240, 241). Next-generation approaches, including SIRPα-Fc fusion proteins with reduced RBC binding and bispecific antibodies, are currently undergoing optimization to further improve the therapeutic index (242, 243).

NKG2A and LILRB checkpoint modulators are also under investigation. NKG2A blockade, exemplified by monalizumab, aims to relieve HLA-E–mediated inhibition of NK and T cells, thereby restoring anti-tumor immunity. LILRB1/2 (ILT2/ILT4) antagonists target myeloid and NK cell checkpoints, which are often exploited by tumor cells to suppress immune responses. Early-phase data in hematologic malignancies are investigating the combinatorial logic of these agents with T-cell or NK cell-based therapies to enhance their efficacy.

Metabolic inhibitors are being developed to relieve immunometabolic suppression within the TME. The IDO pathway, arginase (ARG) pathway, and adenosine pathway are key targets. IDO1 inhibitors aim to reverse tryptophan depletion and kynurenine accumulation, which suppress T-cell function. ARG inhibitors or supplementation strategies seek to restore arginine levels, essential for T-cell activation and proliferation. The adenosine pathway, involving ectonucleotidases CD39 and CD73 and the A2A receptor (A2AR), creates an immunosuppressive environment by converting ATP to adenosine. A2AR antagonists, such as EXS21546, are in clinical trials, with efforts focused on identifying gene signatures that predict patient responses to these inhibitors, thereby increasing the likelihood of trial success (244). The status of hematologic trials for these metabolic modulators is ongoing, with a strong emphasis on translational biomarker considerations to guide patient selection and optimize treatment.

Rational combinations and sequencing are paramount for maximizing therapeutic benefit while minimizing toxicity. The principles often involve a multi-pronged approach (1): mobilizing or dislodging leukemic cells from protective niches using agents like CXCR4 or E-selectin inhibitors; (2) debulking or killing tumor cells with conventional chemotherapy, venetoclax, FLT3i, or IDHi; (3) relieving immune suppression through checkpoint or metabolic blockade; and (4) consolidating immune control with CAR T-cells, BiTEs, or maintenance therapies. Careful consideration of timing windows is essential to minimize myelosuppression and avoid antagonistic signaling pathways. Measurable residual disease (MRD)-guided adaptation of therapy is increasingly used to tailor treatment intensity and sequencing. Known synergistic pairs, such as HMAs with checkpoint inhibitors or VEN with FLT3i/IDHi, are being actively studied, but caution is warranted where overlapping toxicities are likely. These microenvironment-targeted approaches including CXCR4 antagonism, E-selectin blockade, CD47 inhibition, TIM-3/NKG2A/LILRB checkpoint modulation, and metabolic interventions (**Table 1**) are being evaluated to overcome niche-mediated resistance.

**Table 1:** Microenvironment-targeting agents in leukemias.

Pathway / target	Leukemia entity	Therapeutic agent	Evidence level*	Trial Phase / status	Clinical efficacy data	Key toxicities	Clinical implication	Verified NCT ID / trial status
CD47–SIRPα	AML, MDS	Magrolimab (5F9)	Moderate	Phase 3 ongoing (newly diagnosed TP53-mutant AML)	ENHANCE-2 Phase 3: primary outcome pending; prior Phase 1b/2 data showed 88% ORR with azacitidine combination	On-target anemia (manageable with priming and dose escalation); infusion reactions; immune-related adverse events	Disrupts “don’t eat me” signaling; enhances macrophage-mediated phagocytosis; targets TP53-mutant AML with high unmet clinical need	NCT04778397 (Phase 3 ENHANCE-2; ACTIVE, recruiting)
TIM-3	AML, MDS	Sabatolimab (MBG453)	Moderate	Phase 1b ongoing (multi-arm dose escalation with HMA combinations)	Phase 1b: immune activation (↑CD69, ICOS, CD28, 4-1BB; ↓TIGIT, LAG-3, CD160); ~40% disease-response signals; n≈25; follow-up ongoing	Immune-related adverse events (GVHD-like but manageable); reversible laboratory abnormalities	Relieves T-cell checkpoint inhibition; restores CD8+ effector T-cell function; synergistic with decitabine or azacitidine	NCT03066648 (Phase 1b; ACTIVE for AML/MDS)
E-selectin	AML (R/R)	Uproleselan	Moderate–High	Phase 3 ongoing (R/R AML with chemotherapy)	Phase 2a: 65% ORR (uproleselan + MEC) vs 45% (MEC alone); p<0.001; severe mucositis reduced from 58% to 12%; median follow-up 14.8 months	Neutropenia (manageable; improved vs historical controls); occasional gastrointestinal effects; improved tolerability profile	Blocks adhesion-mediated dormancy; disrupts vascular niche; mobilizes quiescent blasts to enhance chemotherapy sensitivity	NCT03616470 (Phase 3 registration trial; ACTIVE, recruiting)
CXCR4	AML	Plerixafor	Moderate	FDA-approved for HSC mobilization; Phase 1/2 AML exploratory trial closed	FDA approval: ≥90% CD34+ mobilization for HSC; Phase 1/2 AML showed partial responses in plerixafor + clofarabine combinations (trial closed early)	Splenic enlargement; neutropenia; risk of rapid leukemic proliferation if mobilized cells not immediately treated	Disrupts CXCL12–CXCR4 niche retention; mobilizes leukemic stem cells from protective microenvironment; AML development halted after early trials	NCT01160354 (Phase 1/2 exploratory; CLOSED; FDA-approved indication for HSC mobilization)
MCT1	AML	AZD3965	Low	Phase 1 solid tumors (DLBCL, Burkitt); preclinical AML studies	Phase 1 solid tumors: MTD defined; partial responses observed; preclinical AML: inhibits lactate export and sensitizes leukemic cells to venetoclax	Reversible laboratory abnormalities; QT monitoring required; manageable gastrointestinal effects	Blocks metabolic adaptation in hypoxic niches; disrupts lactate-driven immunosuppression; AML clinical development pending	NCT01791595 (Phase 1 solid tumors; ACTIVE; no registered AML trials)

This table summarizes agents discussed in Section 8 that modulate bone marrow niche adhesion/trafficking, macrophage checkpoints, T−cell/NK checkpoints, and metabolism. Trial phases and evidence are shown where specified; otherwise “not specified.”

High: Phase 2–3 randomized controlled trials (≥30 patients per arm) or regulatory approval in the target leukemia with established clinical benefit.

Moderate–High: Phase 1b or early Phase 2 data (≥20–30 patients) showing robust efficacy signals and advancement toward Phase 3.

Moderate: Phase 1b trials (≈15–20 patients) or strong observational cohorts with measurable clinical activity and solid mechanistic rationale.

Low–Moderate: Strong preclinical evidence with very limited human data; early Phase 1 exploration only.

Low: Preclinical evidence only; no published clinical trials in the target leukemia.

AML, acute myeloid leukemia; MDS, myelodysplastic syndromes; CML, chronic myeloid leukemia; LSC, leukemic stem cell; HSC, hematopoietic stem cell; HMA, hypomethylating agent; VEN, venetoclax; A2AR, adenosine A2A receptor; CXCR5, C-X-C Motif Chemokine Receptor 5; CD47, Cluster of Differentiation 47; TIM3, T-cell Immunoglobulin and Mucin-domain containing-3; IDO, Indoleamine 2,3-dioxygenase; AML, Acute Myeloid Leukemia; CML, Chronic Myeloid Leukemia; CXCL12, C-X-C Motif Chemokine Ligand 12.

### 8.4 Biomarkers and MRD

The integration of TME biomarkers into measurable residual disease (MRD) assessment is crucial for refining risk stratification, predicting therapeutic response, and guiding therapy sequencing in hematologic malignancies. Predictive and prognostic TME biomarkers encompass various aspects of the microenvironment. The immune contexture, including CD8+ T-cell infiltration, exhaustion states (e.g., expression of PD-1, TIM-3, TIGIT), the abundance of regulatory T cells (Tregs), and NK cell dysfunction, can indicate the likelihood of response to immunotherapies and predict relapse risk (245, 246). Soluble markers, such as soluble PD-L1 (sPD-L1), soluble MHC class I polypeptide-related sequence A/B (sMICA/B), and soluble CD163, have also been linked to relapse risk and disease progression (247).Key TME-linked biomarkers with established or emerging roles in MRD risk assessment across different hematologic malignancies are summarized in **Table 2**.

**Table 2:** TME-linked biomarkers for MRD risk.

Biomarker	Cellular source	Leukemia entity	Clinical utility	Evidence level*	Association with outcomes	Suggested clinical use	References
PD-L1 (blast / immune cell)	Leukemic blasts, myeloid cells, endothelium	AML (esp. FLT3-ITD+), CLL, ALL	Prognostic; Predictive	Moderate–High	Elevated PD-L1 associated with impaired CD8+ T-cell function, reduced chemotherapy response, and inferior overall survival. In FLT3-ITD+ AML, high PD-L1 correlates with adverse outcomes. Soluble PD-L1 levels associate with relapse risk post-remission.	Stratify patients for PD-1/PD-L1 inhibitor–based combinations (often with HMA); monitor immune activation during therapy; incorporate soluble PD-L1 into relapse-risk assessment	(89)
TIM-3	Exhausted T cells, leukemic blasts, monocytes	AML, CLL, ALL	Predictive	Moderate	High TIM-3 co-expression with PD-1/LAG-3/TIGIT indicates severe T-cell exhaustion and poorer survival. Reduction of TIM-3 expression after therapy (e.g., HMA + sabatolimab) correlates with immune recovery. TIM-3 on blasts associates with impaired anti-leukemic immunity.	Identify candidates for TIM-3 blockade combinations; assess depth of T-cell exhaustion; monitor immune restoration during therapy; integrate with PD-1/LAG-3 for exhaustion scoring	(83, 87, 88)
CD47	Leukemic blasts, erythrocytes	AML, MDS, CLL	Prognostic; Therapeutic target	Moderate–High	High CD47 expression confers macrophage immune evasion and correlates with poor prognosis and chemoresistance. TP53-mutant AML shows particularly high CD47 expression and enhanced response to CD47 blockade.	Guide selection for anti-CD47 therapy (magrolimab); refine risk stratification in TP53-mutant AML; monitor on-treatment CD47 modulation and anemia risk	(71, 72)
IDO1	Stromal fibroblasts, dendritic cells, myeloid cells	AML, CLL, ALL, MDS	Immune suppression marker	Moderate	Increased IDO1 activity leads to tryptophan depletion, Treg expansion, and T-cell dysfunction. Elevated IDO1 correlates with IFN-γ–driven immunosuppression and reduced checkpoint inhibitor efficacy.	Identify immunosuppressive niches; guide enrollment into IDO1-inhibitor combination trials; monitor kynurenine:tryptophan ratio as pharmacodynamic surrogate	(135–138)
Adenosine (CD39/CD73 axis)	Tregs; myeloid-derived cells	AML, CLL, ALL, MDS	Immune suppression marker	Moderate–High	High adenosine signaling and CD39/CD73 expression correlate with impaired T-cell proliferation, reduced NK-cell cytotoxicity, checkpoint resistance, and adverse prognosis.	Select patients for adenosine-pathway inhibitors; identify adenosine-rich BM niches; support multi-checkpoint combination strategies	(127–133)
Regulatory T cells (CD4+FOXP3+)	Bone marrow–enriched CD4+FOXP3+ cells	AML, CLL, ALL, CML	Prognostic	Moderate	High BM Treg frequency (>15–20% of CD4+ T cells) associates with inferior survival, early relapse, and reduced checkpoint inhibitor efficacy. Decrease during therapy correlates with immune recovery.	Assess baseline immunosuppressive burden; predict need for combination immunotherapy; monitor immune reconstitution during treatment	(99, 100)
E-selectin	Bone marrow endothelial cells	AML (ND, R/R), ALL	Prognostic; Therapeutic target	Moderate–High	High endothelial E-selectin and blast sLeX expression correlate with adhesion-mediated chemoresistance and relapse. E-selectin blockade improves response and reduces mucositis.	Guide uproleselan use in R/R AML; identify vascular niche–mediated resistance; integrate with spatial profiling	(53, 58) NCT03616470
T-cell exhaustion score (PD-1+TIM-3+LAG-3+TIGIT)	CD8+ T cells (BM-enriched)	AML, CLL, ALL	Predictive	Moderate–High	Co-expression of ≥3 inhibitory receptors marks severe exhaustion and poor survival. Reduction in checkpoint expression post-therapy predicts durable response.	Select candidates for multi-checkpoint blockade; monitor immune recovery; guide sequencing of immunotherapies	(81, 216, 217)

Biomarkers listed reflect Section 8. Platforms and evidence context are provided where specified; otherwise “not specified.” Directionality indicates association with outcomes (e.g., resistance, relapse risk, protection). Suggested uses emphasize risk stratification, therapy selection/sequencing, and integration with MRD.

High – Large prospective cohorts (≥50–100 patients) with consistent outcome associations and independent validation; strong mechanistic support.

Moderate–High – Phase 1b/early Phase 2 trials or observational cohorts (≥30–50 patients) with prognostic or predictive associations and strong mechanistic rationale.

Moderate – Smaller cohorts (15–30 patients) plus mechanistic studies; preliminary prospective validation.

Low–Moderate – Predominantly preclinical data with limited human correlation.

Low – Preclinical evidence only.

MRD, measurable residual disease; IHC, immunohistochemistry; NGS, next-generation sequencing; PD-L1, Programmed Death-Ligand 1; CXCL12, C-X-C Motif Chemokine Ligand 12; MCT4, Monocarboxylate Transporter 4; CD39, Cluster of Differentiation 39; CD73, Cluster of Differentiation 73; ELISA, Enzyme-Linked Immunosorbent Assay; RNA, Ribonucleic Acid; AML, Acute Myeloid Leukemia; CLL, Chronic Lymphocytic Leukemia; ALL, Acute Lymphoblastic Leukemia.

Stromal, adhesion, and metabolic markers provide further insights. CXCL12-high niches, expression of E-selectin and vascular cell adhesion molecule 1 (VCAM-1), and VLA-4 activation signatures are indicative of leukemic cell retention and protection from therapy (248). The presence of CD39/CD73-adenosine hubs signifies an immunosuppressive metabolic milieu (249). Markers like monocarboxylate transporter 4 (MCT4) and carbonic anhydrase IX (CAIX) reflect lactate production and acidosis, which contribute to an immunosuppressive and pro-tumorigenic TME (127). Spatial proximity of LSC signatures to vascular or endosteal regions within the bone marrow can also predict resistance and relapse (250).

The advent of multi-omics approaches is revolutionizing MRD assessment. Integrating conventional flow cytometry and next-generation sequencing (NGS) for MRD detection with single-cell RNA sequencing (scRNA-seq), T-cell receptor sequencing (TCR-seq), and spatial transcriptomics/proteomics allows for a more comprehensive understanding of the TME and leukemic cell heterogeneity (251). This multi-omics integration can refine risk stratification, identify novel therapeutic targets, and guide personalized therapy sequencing. However, standardization and validation of these complex assays are critical, and limitations such as sampling bias, challenges with decalcification of bone marrow samples, and temporal drift in biomarker expression must be carefully considered (252).

#### 8.4.1 Disease-specific nuance

In AML, VEN+HMA regimens serve as a TME-sensitive backbone, with FLT3i/IDHi combinations further addressing specific genetic drivers and their TME interactions. E-selectin and CXCR4 targeting are actively pursued to overcome adhesion-mediated resistance, and TIM-3 development, particularly with sabatolimab, is a key area of investigation (253). CD47 combinations, especially with azacitidine, show promise in high-risk AML (254). MRD assessment in AML is increasingly tied to OXPHOS niches and adhesion markers, reflecting the metabolic and adhesive dependencies of residual leukemic cells (255).

For ALL, the efficacy of CAR T-cell and BiTE therapies is often tempered by barriers within the bone marrow and central nervous system (CNS) niches, necessitating strategies to improve trafficking and overcome immunosuppression (256). Checkpoint blockade has shown variable activity, but MRD-adapted immunotherapy sequencing is becoming a standard of care, particularly in B-ALL, to guide post-remission therapy (257).

In CLL, the era of BTK and BCL2 inhibitors has profoundly reshaped T-cell states and humoral dysfunction (258). Strategies targeting CD47, as well as adenosine and arginase pathways, are being investigated to further improve outcomes (259). MRD assessment in CLL integrates with the unique biology of lymph node proliferation centers, which serve as critical niches for leukemic cell survival and expansion (260).

**Figure 5:**
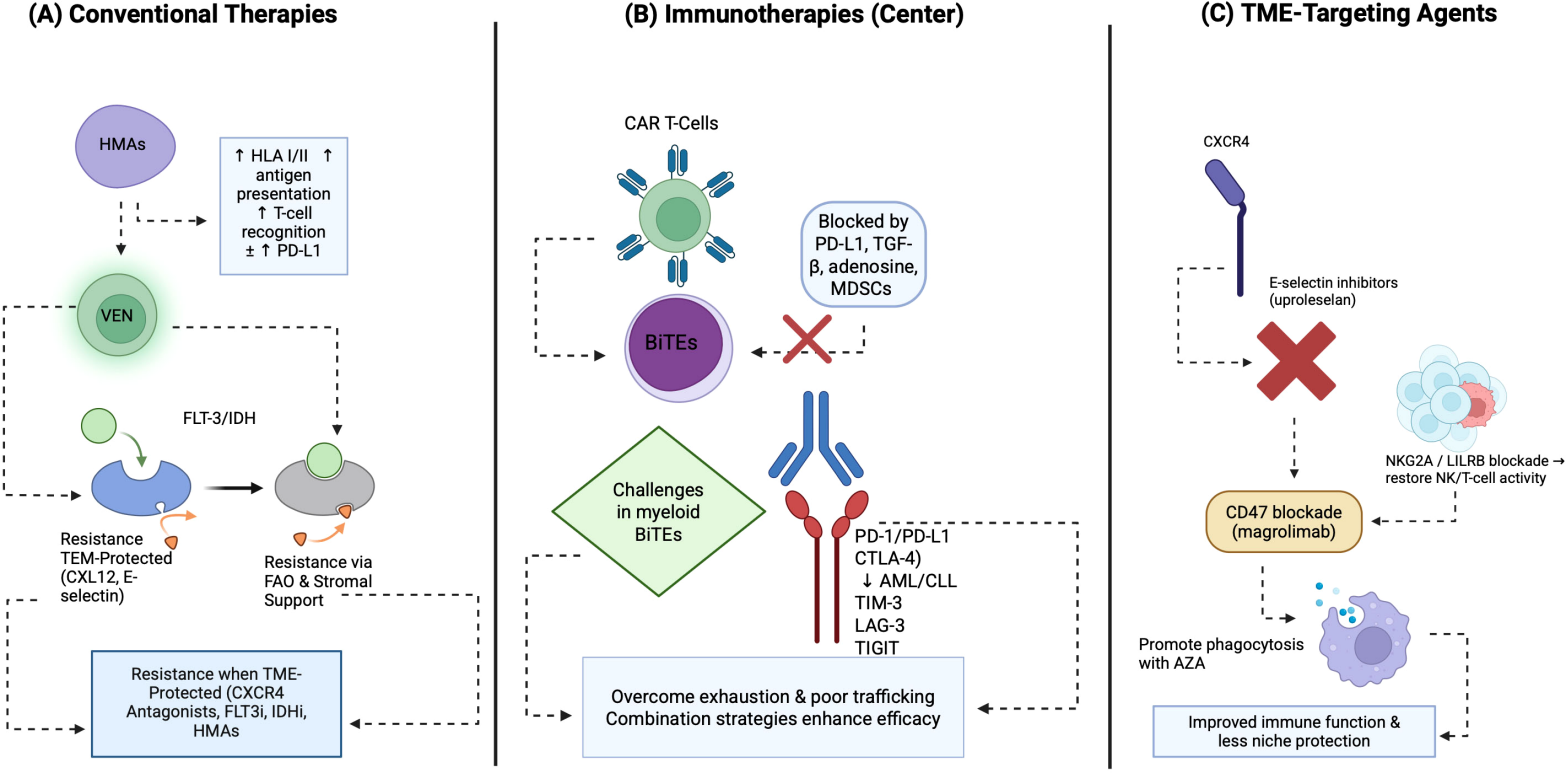
Therapeutic strategies targeting the leukemic tumor microenvironment. **This** figure highlights therapeutic approaches that reshape or disrupt the leukemic niche. **(A)** Conventional therapies such as hypomethylating agents (HMAs), venetoclax (VEN), and FLT3/IDH inhibitors enhance antigen presentation and T-cell recognition, but face resistance mediated by stromal support, fatty acid oxidation, and TME protection (e.g., CXCL12, E-selectin). **(B)** Immunotherapies—including CAR T cells, BiTEs, and checkpoint inhibitors—are hindered by immunosuppressive barriers such as PD-L1, TGF-β, adenosine, and MDSCs. Combination approaches can overcome T-cell exhaustion, improve trafficking, and enhance efficacy, particularly in myeloid leukemias. **(C)** TME-targeting agents directly disrupt protective niches: CXCR4 and E-selectin inhibitors mobilize leukemic cells, while CD47 blockade (e.g., magrolimab) restores phagocytosis and synergizes with HMAs. Additional strategies, including NKG2A/LILRB inhibition, restore NK/T-cell activity and improve immune function. HMA, Hypomethylating Agents; VEN, Venetoclax; FLT3, Fms-like Tyrosine Kinase 3; IDH, Isocitrate Dehydrogenase; TME, Tumor Microenvironment; CXCL12, C-X-C Motif Chemokine Ligand 12; CAR T cells, Chimeric Antigen Receptor T cells; BiTEs, Bispecific T-cell Engagers; PD-L1, Programmed Death-Ligand 1; TGF-β, Transforming Growth Factor Beta; MDSCs, Myeloid-Derived Suppressor Cells; CXCR4, C-X-C Chemokine Receptor Type 4; NK, Natural Killer; LILRB, Leukocyte Immunoglobulin-Like Receptor subfamily B.

For CML, tyrosine kinase inhibitors (TKIs) have normalized disease, but niche-dependent LSCs persist, posing a challenge for achieving treatment-free remission (TFR) (261). CXCR4 re-expression under TKI therapy is a recognized mechanism of LSC persistence (262). Immune checkpoint and metabolic modulators are being assessed to target these residual LSCs and optimize the rates of TFR (263). And the **Figure 5** provides a comprehensive overview of these therapeutic strategies, illustrating how different drug classes intersect with the key TME-driven mechanisms of resistance and immune evasion. This integrated view highlights the rationale for combination therapies designed to simultaneously disrupt multiple axes of the leukemic niche.

## 9 Discussion

The bone marrow tumor microenvironment (TME) is now recognized as a central orchestrator of leukemia pathogenesis, influencing disease initiation, progression, therapy response, and relapse. This review consolidates current insights from a niche-centric perspective, emphasizing the dynamic role of the TME, which leukemic cells exploit to sustain survival, evade immunity, and resist treatment.

The bone marrow niche comprises mesenchymal stromal cells (MSCs), osteolineage cells, endothelial cells, adipocytes, and immune populations such as macrophages, dendritic cells, NK cells, regulatory T cells (Tregs), and effector T cells. Leukemic cells remodel these compartments: they direct stromal differentiation toward osteoblasts, driving drug resistance via Wnt dysregulation and supporting leukemia stem cell (LSC) maintenance in acute myeloid leukemia (AML) (264, 265). Endothelial cells are similarly co-opted; apelin signaling promotes clonal expansion, while angiocrine factors CXCL12 and VCAM-1 sustain B-cell acute lymphoblastic leukemia (B-ALL). Adipocytes, particularly in extramedullary tissues, supply metabolic substrates, linking obesity to poor prognosis (266, 267). Collectively, these elements form a pro-leukemic ecosystem.

Adhesion and trafficking are crucial for leukemic cell retention in protective niches. The CXCL12–CXCR4 axis mediates perivascular dormancy and survival, while antagonists such as plerixafor mobilize cells and enhance chemosensitivity. VLA-4–VCAM-1 and integrin–selectin interactions also support adhesion and NF-κB–driven chemoresistance. Extramedullary sites further act as reservoirs of therapy-resistant cells (268).

Immune evasion is another hallmark of TME-mediated progression. Leukemic cells induce T-cell exhaustion through inhibitory receptors (PD-1, TIGIT, LAG-3, TIM-3), regulated by transcription factors such as TOX and NR4A. Tregs reinforce immune tolerance, while therapies including tyrosine kinase inhibitors (TKIs), BTK inhibitors, BCL2 inhibitors, and hypomethylating agents (HMAs) modulate Treg activity. Impaired antigen processing further blunts immune recognition. Understanding these processes is essential for addressing metabolic adaptations that exacerbate therapeutic resistance.

Metabolic and hypoxic reprogramming intensify immune suppression. HIF-1α pathways elevate monocarboxylate transporters and carbonic anhydrases, promoting survival under hypoxia and inducing lactate accumulation, adenosine signaling, arginine depletion, and IDO1 activity—all dampening cytotoxic immunity. These adaptations sustain leukemic survival and fortify immune evasion (258, 269).

Convergent pathways—including PI3K/AKT/mTOR, Ras/Raf/MEK/ERK, JAK/STAT, and NF-κB—integrate stromal and immune signals (e.g., IL-6, TNF-α). Galectin-3, tumor-derived extracellular vesicles, and immune checkpoint molecules such as B7-H3 enhance adhesion, proliferation, and drug resistance. Advances in spatial, single-cell, and multi-omics technologies have transformed TME characterization. scRNA-seq and TCR-seq delineate immune exhaustion trajectories, while spatial transcriptomics and imaging mass cytometry reveal niche remodeling and metabolic gradients predictive of therapeutic response (270, 271). These tools now permit precise mapping of LSC localization and TME heterogeneity.

Therapeutic implications are substantial. HMAs upregulate antigen-processing genes to re-prime T cells, while venetoclax exploits AML dependence on oxidative phosphorylation (OXPHOS) in hypoxic niches. Immunotherapies such as CAR T cells and bispecific T-cell engagers (BiTEs) face TME-mediated suppression, emphasizing the need for improved strategies. Checkpoint inhibitors show limited efficacy alone but synergize with HMAs in AML and myelodysplastic syndromes (MDS) (84). Microenvironment-targeting agents directly disrupt protective mechanisms: CXCR4 antagonists (plerixafor, mavorixafor) mobilize cells; E-selectin inhibitors (uproleselan) block adhesion and enhance chemotherapy; CD47 blockade (magrolimab) restores macrophage phagocytosis. TIM-3 inhibitors (sabatolimab), IDO and arginase pathway inhibitors, and adenosine A2AR antagonists are under study to reverse immune suppression (272).

TME biomarkers are increasingly integrated into measurable residual disease (MRD) assessment. Immune exhaustion markers (PD-1, TIM-3, TIGIT), Treg abundance, soluble PD-L1, adhesion molecules (VCAM-1, E-selectin), metabolic markers (MCT4, CAIX, CD39/CD73), and spatial LSC signatures refine relapse prediction and therapeutic guidance (273, 274). Multi-omics–based MRD approaches enable more comprehensive evaluation of residual disease.

Therapeutic sequencing relies on multi-pronged strategies: mobilizing leukemic cells, debulking with conventional agents, relieving immune suppression, and consolidating immune control. MRD-guided adaptation is gaining traction, employing synergistic regimens such as HMAs with checkpoint inhibitors or venetoclax with FLT3/IDH inhibitors. Disease-specific contexts matter: in AML, VEN+HMA serves as a backbone for niche-targeting strategies; in ALL, CAR T and BiTE therapies encounter TME resistance; in CLL, BTK and BCL2 inhibitors reshape T-cell states, enabling CD47, adenosine, and arginase blockade; and in CML, persistent niche-dependent LSCs under TKI therapy underscore the need for immune and metabolic modulators (275, 276).

Challenges remain. The heterogeneity of both leukemic cells and their microenvironments complicate universal therapy development. Improved preclinical models are essential to replicate human TME dynamics. Standardization and validation of TME biomarkers for MRD integration remain pressing needs.

## 10 Conclusion

The bone marrow TME is a dynamic, multifaceted driver of leukemia biology and therapeutic resistance. Insights into its cellular, metabolic, and immunological interactions—enhanced by single-cell and spatial technologies—are unveiling novel therapeutic vulnerabilities. Microenvironment-targeting agents and biomarker-driven MRD integration hold great promise for overcoming resistance and personalizing therapy. Continued research into these interactions will be pivotal in translating TME biology into improved patient outcomes.
